# Cultural diversity shaped neolithic subsistence in the Carpathian Basin

**DOI:** 10.1038/s41598-025-88541-z

**Published:** 2025-02-04

**Authors:** M. L. C. Depaermentier, M. Kempf, E. Bánffy, K. W. Alt

**Affiliations:** 1 Faculty of History, Vilnius University, Universiteto g. 7, Vilnius, 01513 Lithuania; 2 Quaternary Geology, Department of Environmental Sciences, University of Basel, Bernoullistrasse 32, Basel, 4056 Switzerland; 3 Institute of Archaeology, HUN-REN Research Centre for the Humanities, Budapest, Hungary; 4 Research Unit Budapest, German Archaeological Institute, Romano-Germanic Commission, Budapest, Hungary; 5 Department of Natural and Cultural Human History, Danube Private University, Krems, Austria; 6 Institute for Prehistory and Archaeological Science, Basel University, Basel, Switzerland; 7 Department of Geography, University of Cambridge, Cambridge, United Kingdom; 8 Department of Ancient Civilizations, University of Basel, Petersgraben 51, 4051 Basel, Switzerland

**Keywords:** Carbon stable isotopes, Nitrogen stable isotopes, Environmental modeling, Cluster analyzes, Agropastoral, Neolithization., Environmental impact, Element cycles

## Abstract

Stable carbon and nitrogen isotope analysis from archaeological human and animal bone collagen is a powerful tool to enlighten past socio-agricultural systems and their close links to environmental conditions. To assess whether isotopic patterns are a function of cultural behavior or rather determined by environmental diversity, we test the spatio-temporal effects of conditional and multivariate exploratory and environmental models on stable isotope variability in the Neolithic and Chalcolithic Carpathian Basin (6000–2800 BCE). Our results stress that variation in environmental settings influence both the isotope baselines and the subsistence strategies of early agricultural societies. Major shifts in isotopic patterns are, however, culturally-driven, which is particularly visible with the development and intensification of adapted agropastoral practices from 5500 − 5350 BCE and among the diet between different cultural groups. We conclude that socio-cultural diversity was the main driver of dietary habits and isotopic variability in early agropastoral societies of Neolithic Hungary.

**Supplementary Information:**

The online version contains supplementary material available at 10.1038/s41598-025-88541-z.

## Introduction

**Fig. 1:**
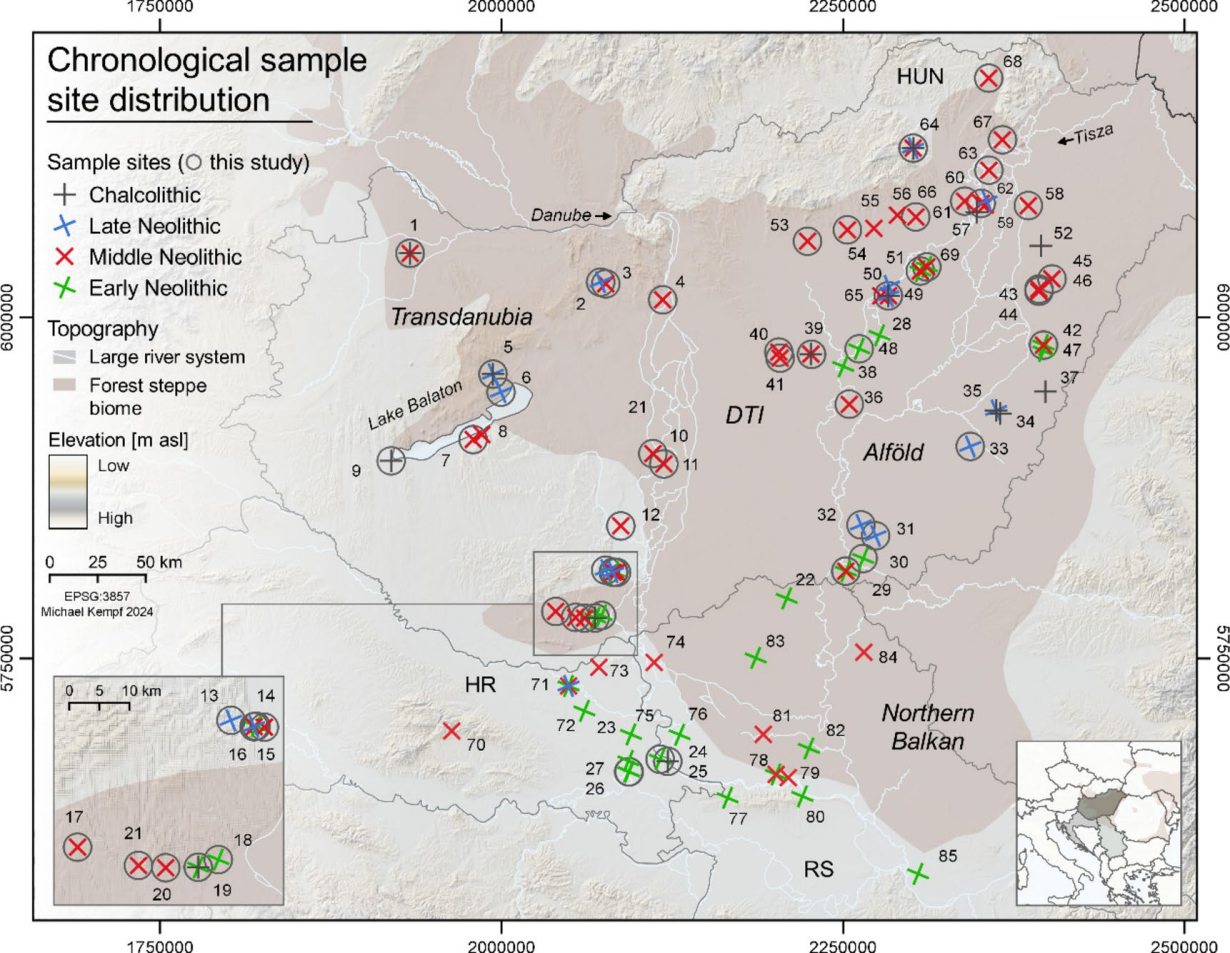
Geographical distribution of the studied sites. Site IDs and reference to the data from the literature are listed in table S1. DTI = Danube-Tisza Interfluve; Modern country borders: HUN = Hungary; HR = Croatia; RS = Serbia. This map is produced using QGIS 3.10.12 (QGIS Geographic Information System. QGIS Association. http://www.qgis.org (2024)).To visualize the site distribution for each period separately, see the figures S1 to S4 as well as the repository to this article^19^.

Stable isotope analyses have proven to be powerful tools to gain new insights into subsistence strategies and dietary habits of past communities^1–3^. This method has been applied to study Neolithic food economies in different parts of present-day Europe including the Carpathian Basin, yet the studies in this area are mostly restricted to site-specific case studies^4–6^ or dedicated to the Neolithic-Chalcolithic transition^7,8^. The potential of this method to track changes and disparities in subsistence strategies thus remains underexploited and a large-scale and diachronic stable isotope study is still missing for the Neolithic Carpathian Basin. This paper fills this gap by presenting the largest dataset of new and compiled results of carbon (C) and nitrogen (N) stable isotope analyses carried out on human and animal bone collagen from present-day Hungary, Croatia, and Serbia dating from the Early Neolithic (EN) to the Chalcolithic (Fig. 1; Table 1, Tab. S1). This large spatio-temporal approach tracks the evolution and the gradual northwards spread of the Neolithic agropastoral way of life, i.e., a subsistence-oriented form of agriculture that combines field cultivation and livestock farming on natural pastures^9–11^.

Starting from around 6000 BCE (Table 1), the first farmers coming from the northern Balkans spread in what is present-day Hungary, the Körös cultural group heading to the east in the Alföld and the Starčevo group to the west in Transdanubia^9^. During the Middle Neolithic (MN), new agricultural techniques enabled both a decisive shift to the actual Neolithic way of life and the expansion towards more diverse habitats^10,12,13^, while new input from the northern Balkans increased the cultural diversity in southern Transdanubia^13–15^. A higher degree of socio-cultural and agropastoral complexity was reached during the Late Neolithic (LN)^16,17^, and considerable changes in settlements, subsistence strategies, and material production marked the transition to the Chalcolithic period, while yet another input from the Balkans impacted Transdanubia^18^. It is nevertheless still difficult to seize to what extent this cultural diversity and the observed transformations in land-use strategies and archaeozoological assemblages are related to specific agricultural, husbandry, and/or dietary practices (see text S1).

To identify and understand subsistence strategies, cultural changes, and population shifts throughout the Neolithic and Chalcolithic periods, it is fundamental to integrate both the cultural dimension and the environmental parameters underlying these processes. It is, however, challenging to assess the influence of both factors in the observed isotope variability. To cope with these challenges, the following research questions were addressed: (i) Are there changes in subsistence strategies over time during the Neolithic and Chalcolithic? (ii) Can we identify similarities or disparities in dietary practices between the contemporaneous and successive cultural groups in this area? (iii) Is it possible to disentangle cultural and environmental drivers in the observed isotopic patterns? We present an innovative analytical workflow that integrates multivariate exploratory modeling and multiproxy explanatory analyses to investigate spatio-temporal effects of cultural versus environmental factors on stable isotope variability in the Neolithic and Chalcolithic Carpathian Basin.

**Table 1:**
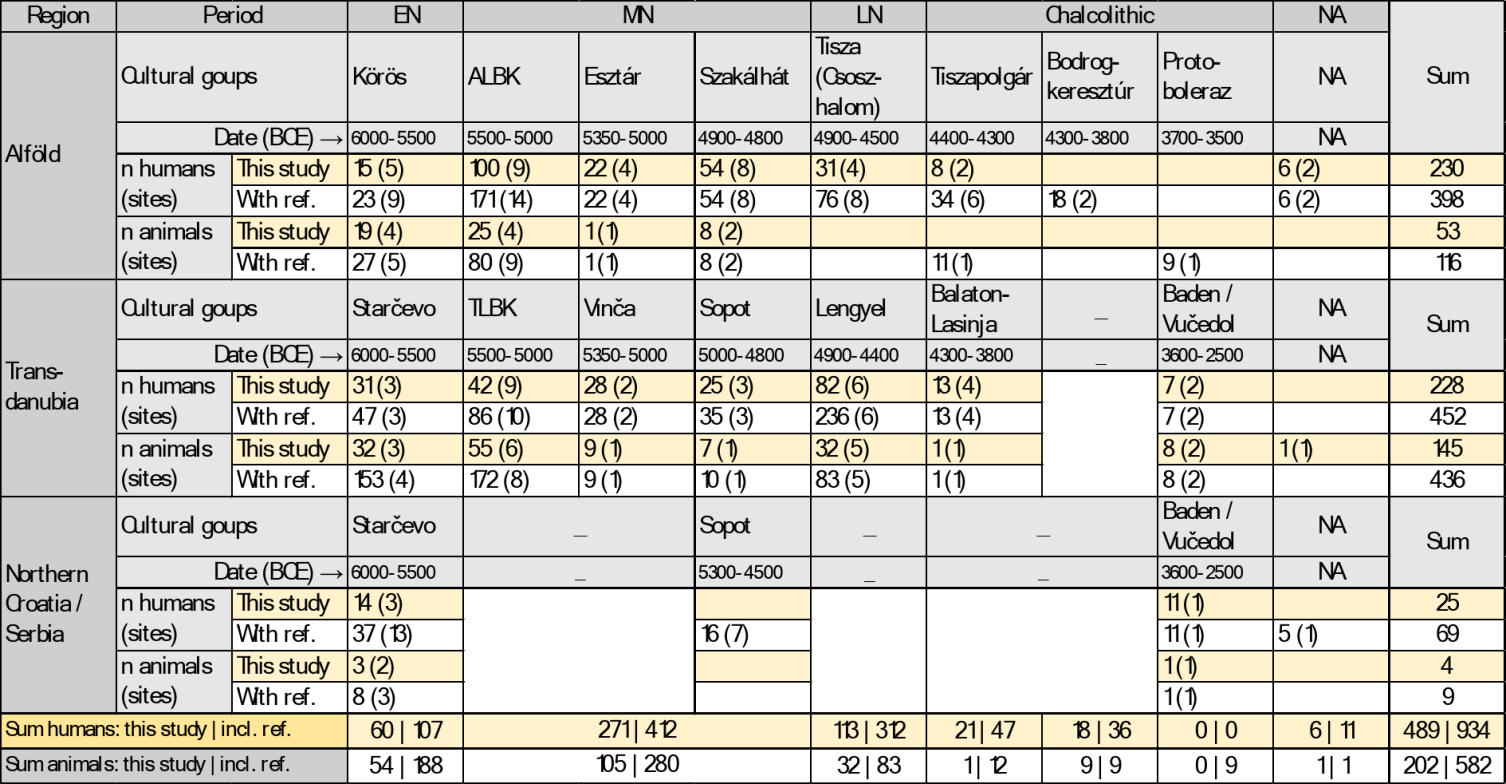
Number of human and animal bone collagen samples.

Total number of samples that provided reliable isotope data, depending on the main region, period, and predominant cultural group. Some analyzed individuals and animals were not associated to any chronology or cultural group and are hence listed here under “NA”. A detailed list of samples including those that did not meet the quality criteria and specifying the reference of the data collected from the literature is available in table S1.

## Results

Here we present the results of stable isotope analyses carried out on human and animal bones from the Neolithic and Chalcolithic Carpathian Basin. Because reliable baselines are lacking at most sites, the relationship between animal and human data will be only expressed in terms of offsets between the mean values at each site providing enough samples of each category. The results will also be presented with respect to the determined environmental clusters. Because cultural and genetic inputs from the northern Balkan have played an important role in both Transdanubia and the Alföld, a comparison dataset from northern Croatia and northern Serbia was included in the study. However, soil data are not available from the northern Balkan region for this study, therefore, these samples are not integrated into the environmental cluster analysis.

### Fauna

When omitting the poorly preserved samples as well as outlier *Bos taurus* (HARG9, with -31.76‰), the δ^13^C values from this study’s dataset (*n* = 194) spans from  -23.98‰ (ovicaprid MEKÖ08) to -18.28‰ (*Bos taurus* BENA16) with a median value at -20.51‰ (1st Qu.: -20.94‰, 3rd Qu.: -20.02‰). The δ^15^N values span from 3.5‰ (ovicaprid KON15) to 11.7‰ (*Canis familiaris* ABO27), with a median value at 7.3‰ (1st. Qu.: 6.5‰, 3rd Qu.: 7.4‰). By including other references and previously published data, the range is extended to from -27.18‰ (fish) to -17.00‰ (*Bos*) for δ^13^C values (1st. Qu.: -20.93‰, median: -20.4‰, 3rd Qu.: -19.9‰) and to 3.2 (*Equus hydruntinus*) for δ^15^N values (1st. Qu.: 6.3‰, median: 7.1‰, 3rd Qu.: 8.1‰) (fig. S5). Only the well represented species (*Bos*, *Sus*, ovicaprids, cervidae) were used for the rest of the analyses.

**Fig. 2:**
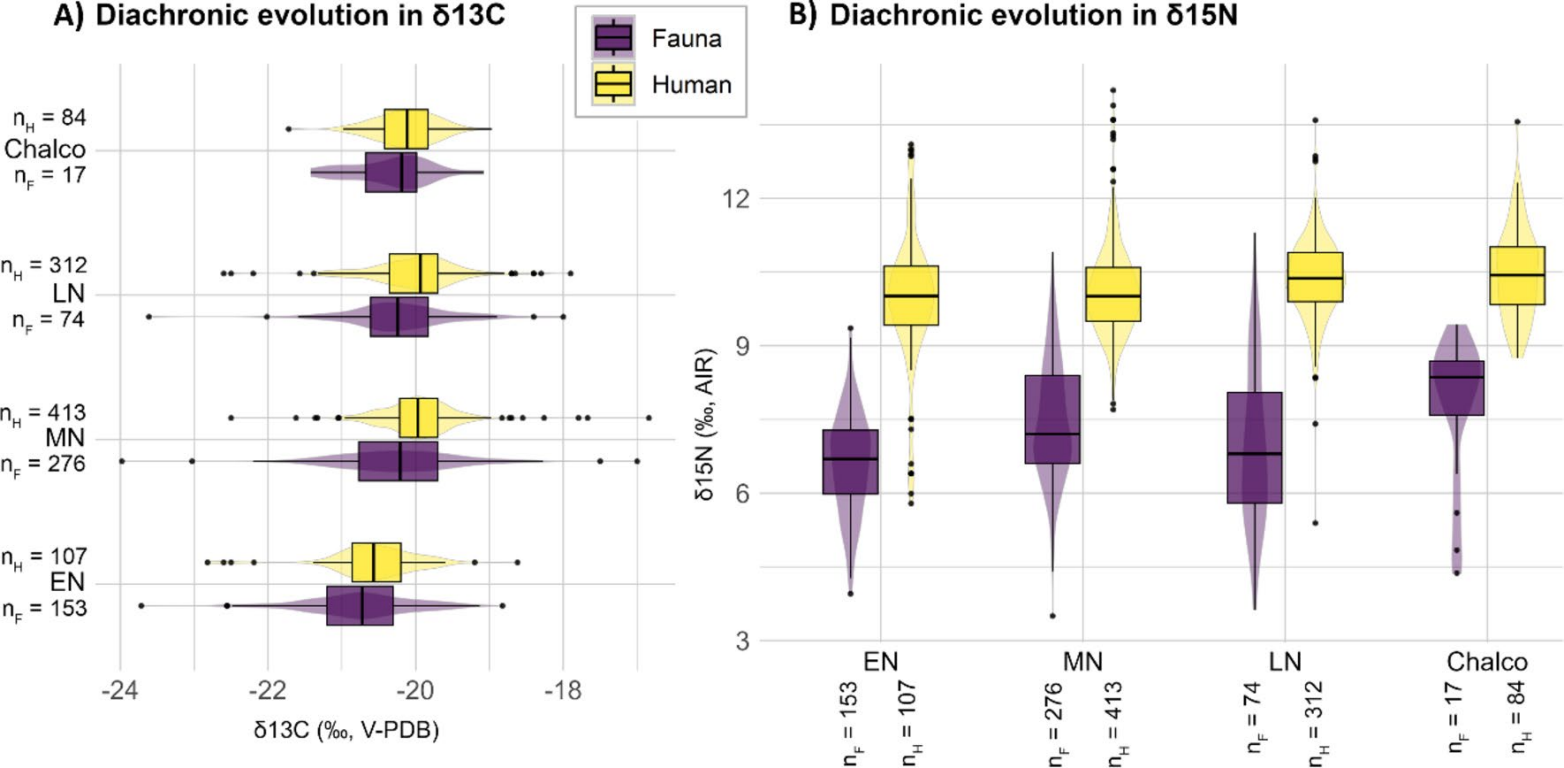
Carbon and nitrogen isotope values over time. Diachronic evolution of δ^13^C (**a**) and δ^15^N (**b**) values among humans and animals (including the omnivores and herbivores only). Detailed overviews of the animal δ^13^C and δ^15^N values over time among the main animal species are available in fig. S6 and fig. S7 respectively.

Diachronically, a slight but statistically significant increase in δ^13^C values among the fauna, except for the ovicaprids (*n* = 10), is further noteworthy from the EN to the LN and particularly marked between the EN and the MN (Fig. 2a; Tab. S2; Fig. S6). An overall increase can be observed in δ^15^N values over time as well (Fig. 2b; tab. S2), but only among the domesticates and with an important drop during the LN, in particular for the *Bos taurus* (*n* = 19) (tab. S2, fig. S7). A drop in LN is also visible for the *Sus scrofa* (*n* = 10), whereas the *Sus domesticus* (*n* = 87) and the ovicaprids (*n* = 124) have increasing δ^15^N values over time (fig. S7). The observed decrease in the cervidae’s δ^15^N values over time is not statistically significant and should be verified with a larger sample size (tab. S2, fig. S7). Spatially, the animal δ^15^N values are more elevated in the Alföld compared to Transdanubia (tab. S2; fig. S8a). On the other hand, the δ^13^C values are overlapping between both main regions (tab. S2; fig S8a). Both δ^15^N and δ^13^C values are slightly lower in southern Hungary compared to northern Hungary (tab. S2; fig S8b). No comparison can be done at the cultural group level due to too small sample sizes.

### Humans

The well-preserved human bone collagen samples from the Neolithic or Chalcolithic periods (*n* = 490) show δ^13^C values spanning from -22.82‰ (TIDO03) to -16.84‰ (BENA06) – although the latter individual has not been radiocarbon dated and the high δ^13^C value may point towards a younger period^20,21^. This also applies at least for the supposedly ALBK juvenile female HAJE20 with a δ^13^C value of -17.79‰. The median δ^13^C value is -20.16‰ (1st Qu.: -20.54‰, 3rd Qu.: -19.83‰). The δ^15^N values presented in this study are spanning from 5.8‰ (TÖSM06) to 13.9‰ (SZEH07), with a median value at 10.1‰ (1st. Qu.: 9.6‰, 3rd Qu.: 10.7‰). Adding previously published data to the dataset hardly modifies the summary statistics for δ^13^C values (1st. Qu.: -20.41‰, median: -20.02‰, 3rd Qu.: -19.73‰) and expands the range of δ^15^N values, now spanning from 5.4 to 14.2‰ (1st. Qu.: 9.7‰, median: 10.2‰, 3rd Qu.: 10.8‰). Even though infants should be considered separately due to potential breastfeeding effects^22^, the life stage is not recorded for each individual and infants might not be recognized in this dataset. When excluding the individuals that were clearly identified as infants, the δ^15^N values nevertheless show similar results than the whole sample (min: 6.0‰, max: 13.60‰, 1st. Qu.: 9.7‰, median: 10.2‰, 3rd Qu.: 10.7‰).

**Fig. 3:**
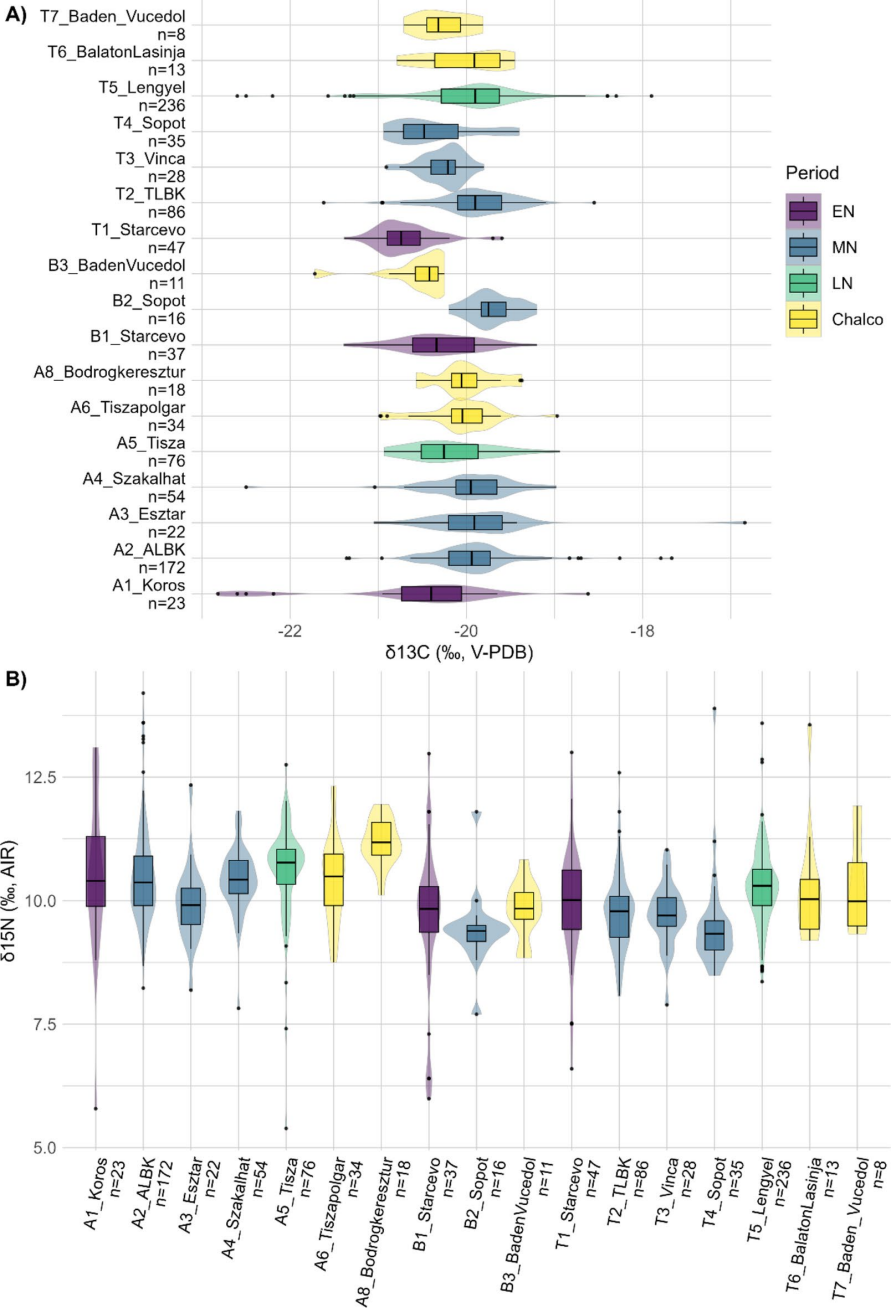
Human carbon and nitrogen isotope values among cultural groups. (**a**) Human δ^13^C values among cultural groups. (**b**) Human δ^15^N values among cultural groups. A = Alföld, B = northern Balkan, T = Transdanubia. The numbering corresponds to the chronological order in each region.

In terms of diachronic evolution, there is a significant increase in δ^13^C values between the EN and the MN (Fig. 2a; tab. S2). This is visible in particular in the Alföld and in the northern Balkans, while in Transdanubia this applies only between the Starčevo and the TLBK groups, whereas δ^13^C values are particularly low at the MN Vinča and Sopot sites (Fig. 3). During the LN, the Transdanubian Lengyel samples exhibit a wide range, whereas the Tisza group in the Alföld shows considerably low δ^13^C values – comparable to the Sopot group.

An increase is also attested in δ^15^N values from the Alföld over time (Fig. 2b; tab. S2) – except for the drop noticed for the Esztár group (*n* = 22) (Fig. 3). The Transdanubian sites show a more varied pattern, with particularly low δ^15^N values for the MN Vinča and Sopot groups. In the northern Balkans, there is an important drop in δ^15^N values between the EN Starčevo and the MN Sopot samples. Just as for animals, human δ^15^N values are slightly more elevated in the Alföld than in Transdanubia but there is hardly any difference in δ^13^C values between both main regions nor is there any visible pattern between southern and northern Hungary in terms of δ^15^N and δ^13^C values (tab. S2; fig. S9).

### Human-fauna-offset

**Fig. 4:**
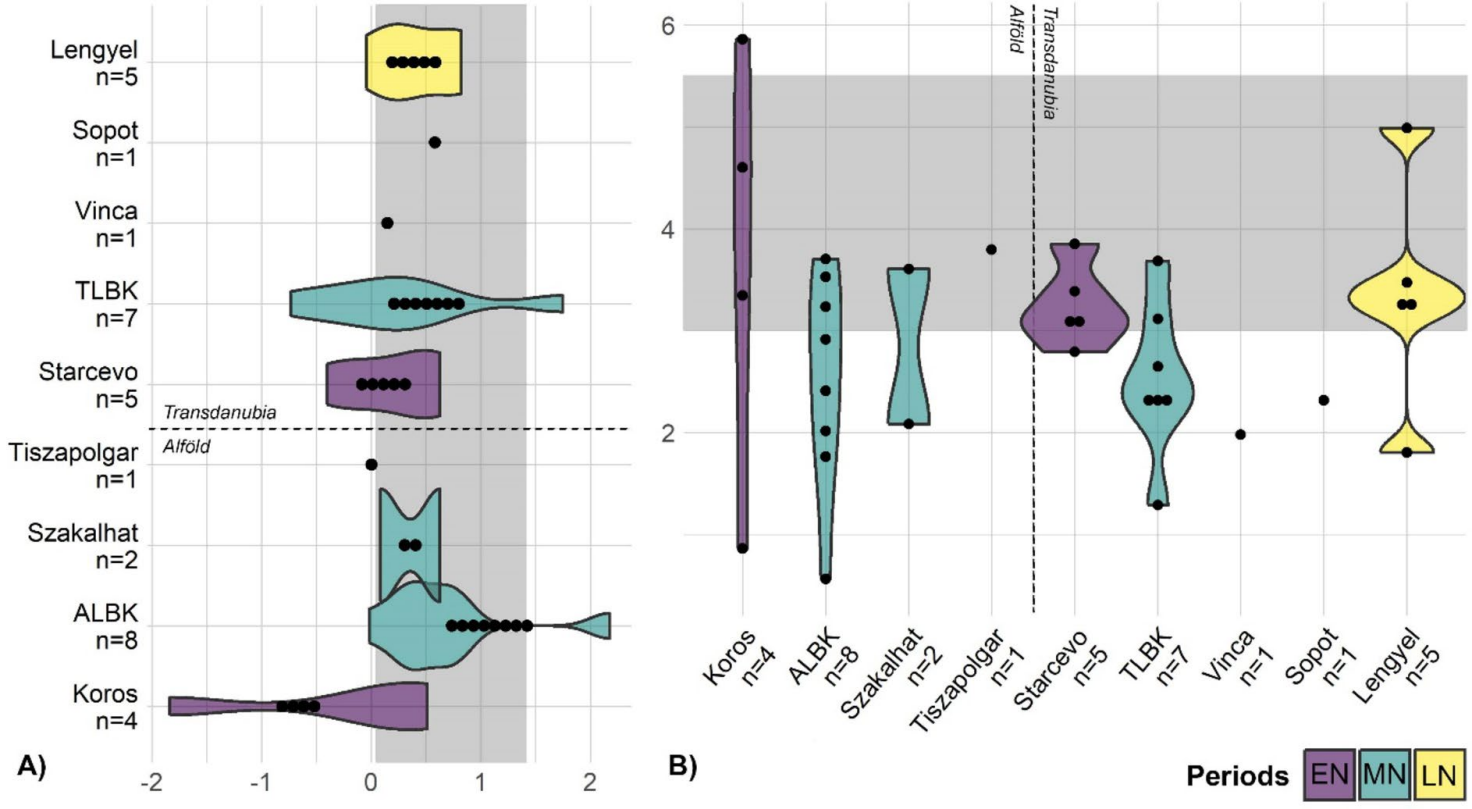
Offsets between human and fauna isotope values. The offsets were calculated at the site level with contemporaneous sample sizes and excluded the carnivores among the animal sample. The gray zones show the expected trophic level offset between human collagen and the consumed animals for both carbon (**a**) and nitrogen (**b**) isotopes. The Vinča and Sopot groups belong to the MN and the Tiszapolgár group to the Chalcolithic.

The offset in δ^13^C values between humans and fauna spans from -1.84 to 2.18‰ (1st Qu.: -0.01‰, median: 0.35‰, 3rd Qu.: 0.63‰) (tab. S3, Fig. 4). The negative offsets and offsets beyond 1‰ would suggest environmental differences in the food resources between humans and the consumed fauna, which is particularly visible during the EN, while the other values may represent a simple trophic level shift. The offset in δ^15^N values varies between 0.6 and 5.9‰ (1st Qu.: 2.3‰, median: 3.1‰, 3rd Qu.: 3.5‰). An offset between 3 and 6‰ represents the expected trophic level^23^, which is visible in the EN and LN, while the offset in the MN is mostly below 3‰.

### Environmental clusters

**Fig. 5:**
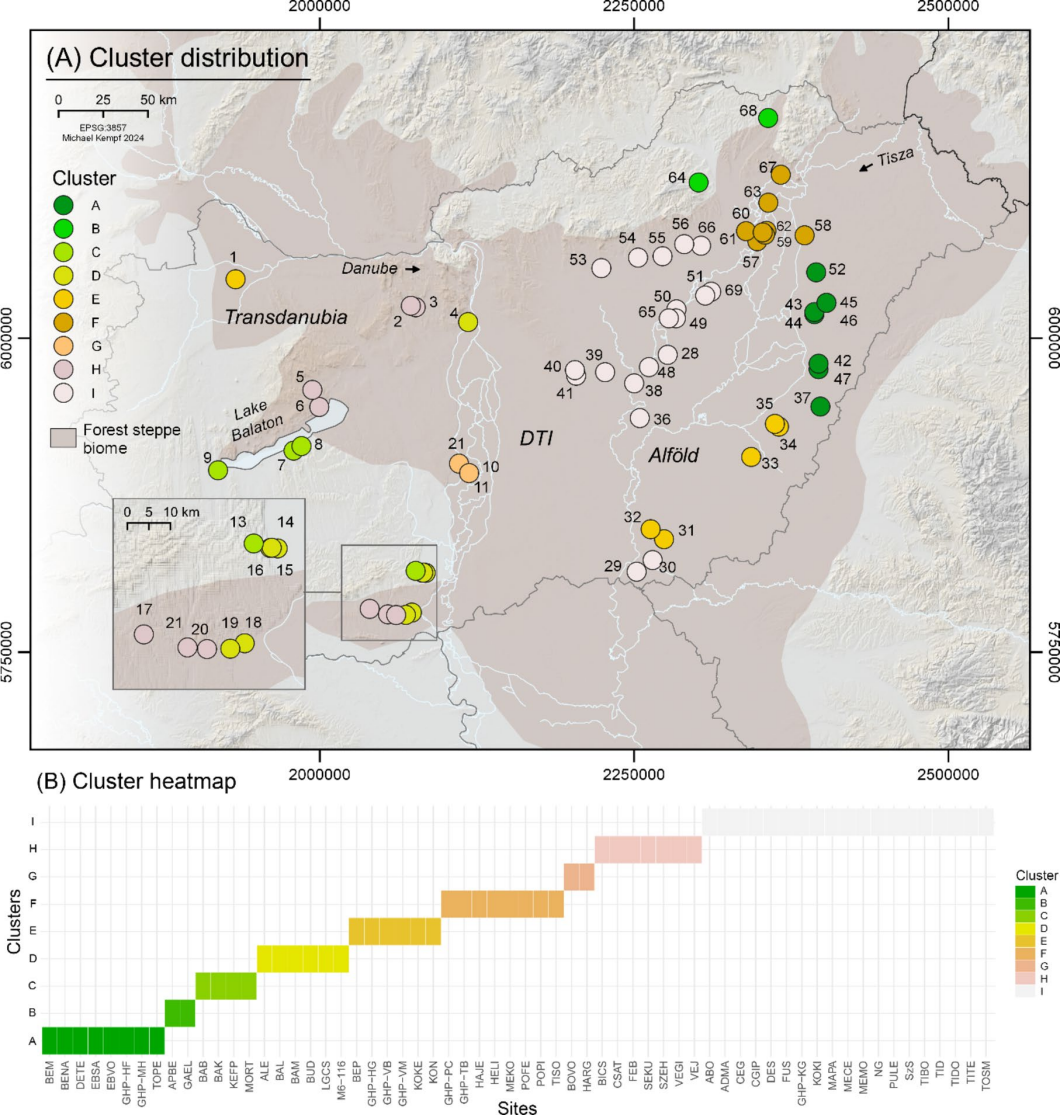
Site cluster distribution (**a**) and heatmap (**b**). K-means (k = 9) site cluster distribution in the study area from drought index (wCON) and soil unit cluster analysis. Site ID and additional information are available from tables S1 and S4 as well as in the repository to this article^19^. This map (**a**) is produced using QGIS 3.10.12 (QGIS Geographic Information System. QGIS Association. http://www.qgis.org (2024)).

**Fig. 6:**
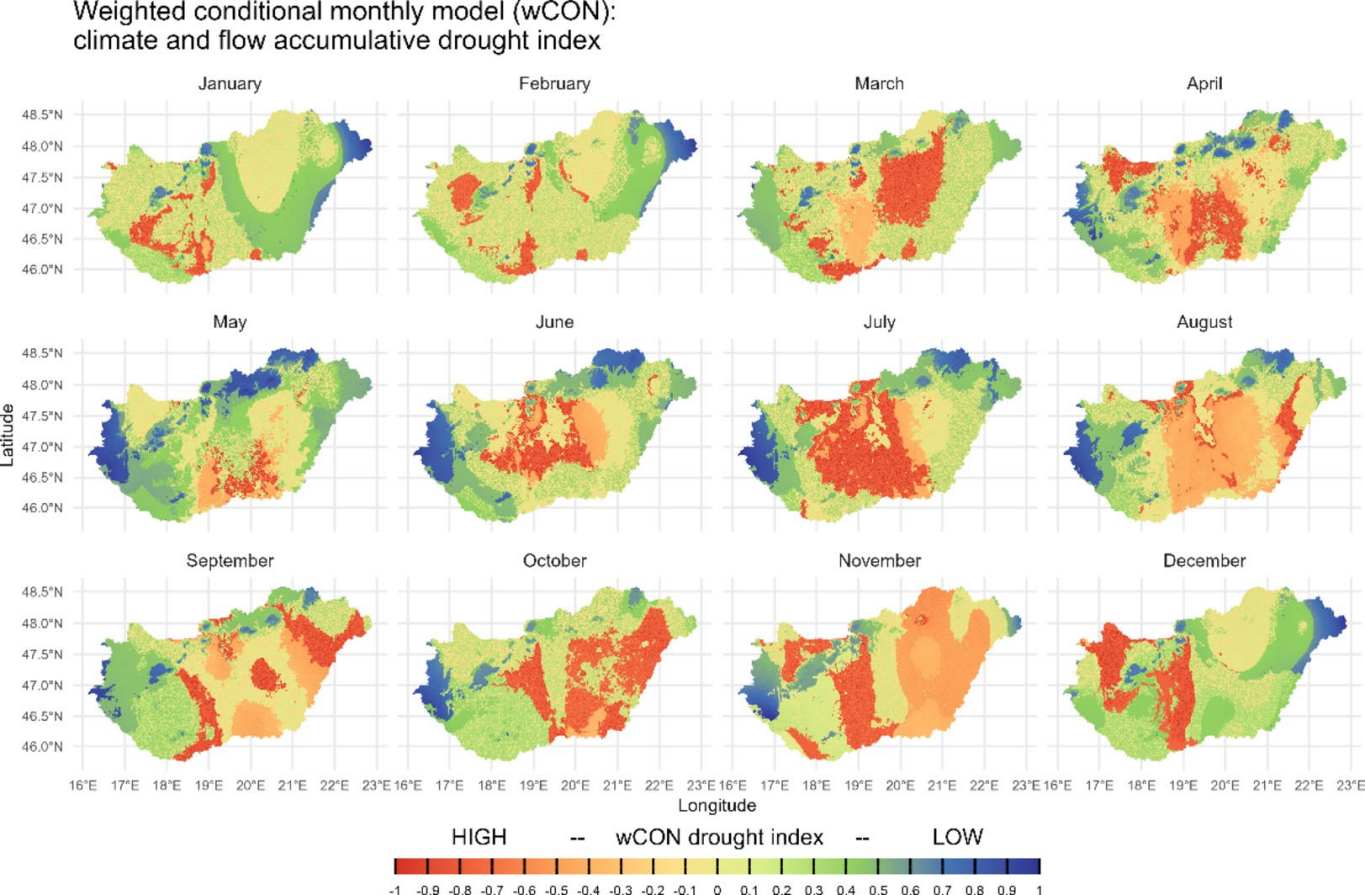
wCON drought index for the Carpathian Basin. wCON model outputs are based on simulated and weighted flow accumulation and the climate conditional model CON using precipitation and temperature monthly values. Low values show strong drought vulnerability, high values indicate humid conditions for each month. The maps were produced by M. Kempf using the R-software 4.3.2 (https://www.r-project.org/)^65^. See the article’s repository for a detailed overview at the site level^19^.

**Fig. 7:**
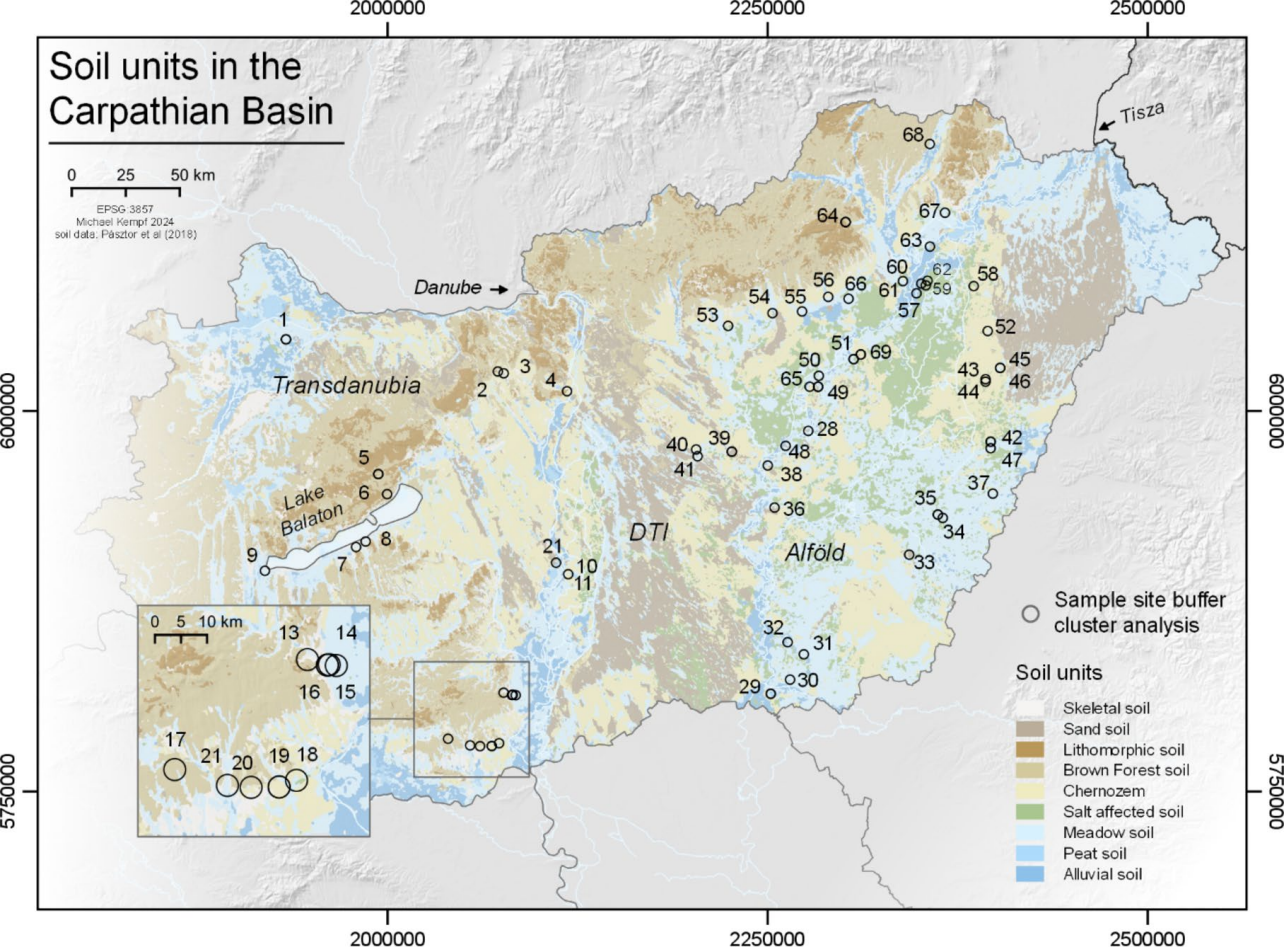
Soil units in the Carpathian Basin. Soil units and site selection buffers (3000 m) for K-means cluster analysis of soil and monthly drought index (wCON) (for soil data, see^24^). Site ID and additional information are available from tables S1 and S4. A detailed overview at the site level is available from the repository to this article^19^. This map is produced using QGIS 3.10.12 (QGIS Geographic Information System. QGIS Association. http://www.qgis.org (2024)).

Cluster analysis (*K-means*, *k* = 9) of the reliable sample was performed using a weighted monthly drought index (wCON) and a soil unit dataset within a 3000 m spatial catchment around each site (Fig. 5). Sites cluster according to regional environmental similarities, based on a combination of climatically weighted flow accumulation, precipitation, temperature (Fig. 6) and soil units (Fig. 7)^19^. The strong monthly drought vulnerability of the Carpathian Basin is regionally mitigated by enhanced superficial streamflow and sub-surface aquifer development, resulting in a meandering and braided river run-off character of the major drainage systems within the basin. High temperatures during summer, however, dominate evaporation rates particularly in the central part of the plain (DTI), leading to increased drought vulnerability. The interplay of sandy soils with low water storage capacity in the DTI and low rainfall and prolonged heatwaves further accelerates drying-up processes. Alluvial and meadow soil units are less affected. We observe a strong zonal differentiation between alluvial and meadow soil site clusters with lower drought vulnerability during the first half of the year (Alföld), along the river Tisza floodplain, and towards Transdanubia, which is affected by both different soil properties as well as hydroclimatic and run-off characteristics.

In general, the site distribution according to the environmental clusters is significantly dependent on the major regions Alföld and Transdanubia (tabs. S2 and S4, Fig. 5 and S10). On the diachronic perspective, the EN sites are dominated by Chernozem or meadow soils (tab. S4), and fall within two clusters for Körös (*n* = 9 sites) and one cluster for Starčevo (*n* = 3 sites) (fig. S11). This represents in each case the most frequent cluster among the cultural groups from each major region – Alföld and Transdanubia, respectively. On the contrary, the MN sites (*n* = 25 in the Alföld and *n* = 14 in Transdanubia) show the greatest diversity in environmental conditions and are distributed over all nine clusters. The LN sites (*n* = 5 in Transdanubia and *n* = 7 in the Alföld), and to a lesser extent the Chalcolithic sites (*n* = 4 sites in Transdanubia and *n* = 9 sites in the Alföld), show a slightly lower diversity in environmental clusters compared to the MN.

When comparing the isotope variability between the clusters (A to I) it is first noteworthy that human and herbivore δ^13^C and δ^15^N values hardly show similar patterns, except cluster F, in which alluvial and salt affected soils are dominant (tab. S4), and in which both the human and animal samples show among the highest δ^15^N values (fig. S12). Another striking result is that the herbivore isotope values are much more varied between clusters than the human values.

## Discussion

Overall, the C isotope composition of human and animal bones shows a terrestrial C_3_-plants-based diet for all regions and periods. The few samples showing δ^13^C values above -18.00‰ could point towards a diet including C_4_ plants, which is however considered unlikely for this period (see text S3) and would require a direct radiocarbon date to verify the affiliation of these samples to the Neolithic or Chalcolithic periods^20,21^. On the other hand, δ^13^C values below -22.00‰, which may indicate a diet derived from forested environments^25,26^, are seldom in both humans (*n* = 8) and animals (*n* = 19, when excluding freshwater fish samples). Among animals, this expectantly mainly concerns the cervidae and to some extent the suids. This suggests that during the Neolithic and Chalcolithic, the food predominantly originated from open environments or fields for both humans and domesticated herbivores (text S3). The difference in δ^15^N values between *Sus scrofa* and *Sus domesticus* moreover indicates that the latter (with δ^15^N values rising above 7.5‰ over time, see fig. S7) probably had access to food residues from the human diet.

### Diachronic dynamics in subsistence strategies

At EN – and especially Körös – sites, humans often exhibit lower δ^13^C values than animals (Fig. 4). This suggests that earliest farmers in this area were possibly including more food from forested or freshwater ecosystems in their diet compared to the later groups^2^. This assumption is supported by the shift in human δ^13^C values between the EN and the MN (especially LBK) (Fig. 2) sites as well as by the archaeozoological records, which show that fishing and hunting were important subsistence activities at many EN/Körös sites^27,28^. It is possible that such activities were still needed to cope with an emerging agricultural system that was still based on Mediterranean species and traditions in both the Alföld and Transdanubia^28–30^. This situation also implied that the spread of EN system was restricted to the warm and dry southern part of the Carpathian Basin, delimited in the north by the so-called Central European Balkan Agro-Ecological Barrier (CEBAEB)^31,32^.

During the MN, new agricultural techniques such as manuring emerged, new crops including pea and opium poppy got integrated, the husbandry strategies got dominated by cattle^10^, and there was an unprecedented demographic increase^33,34^. This triggered a geographical expansion of Neolithic settlements northward as well as towards higher locations and more diverse environmental settings^11,30^. This larger environmental diversity is reflected by the larger spectrum in δ^13^C values compared to the EN sample (Fig. 2). The particularly low offset in δ^15^N values between humans and herbivores/omnivores at MN sites (mostly < 3‰, see Fig. 4) indicates a low protein diet for the humans and suggests that, with this evolution of agricultural practices, crops were playing a more important role than animal products in human diet.

A gradual increase in δ^13^C and δ^15^N values is observed in both human and domesticated animal bone collagen from the EN to the LN/Chalcolithic (Fig. 2). This could theoretically be related to a progressive change in climatic conditions, but the decrease in wild herbivore and omnivore δ^15^N values (fig. S7) as well as the current state of research suggesting a climate shift only after the LN^35^, imply to reject this hypothesis. This data thus highlights an increase in natural or intentional manuring^36,37^. This supports both previous studies^7^ and the archaeological records^38,39^. This can further be put into perspective with the increasing role of salt during this period^40^, as husbandry practices intensified, increasing the demand for salt to satisfy the livestock needs. Intensified land-use strategies may have been a response to the increased food demand implied by the attested demographic boom in the first half of the fifth millennium BC^41^.

The drop in *Bos taurus* δ^15^N values during the LN may, however, highlight a shift in husbandry strategies during that period (fig. S7). Cattle were possibly kept in more extensive and diverse areas without manuring effect, whereas smaller domesticates were kept in smaller and more intensively managed areas, probably closer to the settlements. This is attested by the archaeological records showing larger herds that needed larger grazing areas^42^ – a pattern accompanied by a more extensive settlement distribution^11,17^. The large offset in δ^15^N values between humans and animals from LN sites (Figs. 2 and 4) might reveal an increased role of pastoralism even before the Chalcolithic period, but the reduced mobility rate in the LN^43^ and previous in-depth investigations^7^ do not support this hypothesis. This large offset is therefore another evidence for the consumption of crops coming from manured fields.

### Cultural diversity

Beyond the considerable shift in land-use strategies, subsistence practices, and dietary habits between EN Körös/Starčevo and MN ALBK/TLBK, it is noteworthy that the MN Vinča and Sopot groups in southern Transdanubia are characterized by considerably lower δ^15^N and δ^13^C values in human bone collagen compared to the contemporaneous TLBK sites (Fig. 3). This shows strong differences in agricultural and dietary habits between these contemporaneous cultural groups despite their presence in the same regions. Archaeologically, the Vinča and Sopot groups are known to have strong relationships to the Northern Balkans^44^ (see also text S1). This Balkan origin was confirmed by genetic data^45,46^ and to some extent supported by strontium and oxygen isotope analyses^43^. Yet the considerably higher δ^13^C values observed in the comparison Sopot samples from the northern Balkan (*n* = 16, see Fig. 3) do not suggest that the northern Balkan Sopot dietary habits were kept in Transdanubia – even though differences in environmental settings may have resulted in both different δ^13^C values and subsistence strategies. One hypothesis would be that since the Sopot groups in Transdanubia were sharing their habitat with local TLBK groups, they adapted their diet by relying more on resources not used by TLBK people, such as food from forested and freshwater ecosystems. A very good example of such a coexistence in a restricted space can be found at Alsónyék-Bátaszék^27,47^.

Moreover, and despite their genetically attested contacts with local foragers^45,46^, the Vinča and Sopot groups from both regions show no evidence of a protein-rich diet (Figs. 3 and 4), which would have reflected an important role of hunting and fishing as suggested by their low δ^13^C values (Fig. 3) – the Neolithic freshwater fish consumed in the Carpathian Basin showing also elevated δ^15^N values^5,6^. It is therefore possible that in particular plants and fruits gathered in the forest played an important role in their diet. A more comprehensive isotope baseline would be needed at each site to clarify this assumption. The small number of sites providing isotope data may further imply that these are site-specific rather than cultural-specific patterns^10,15^. This applies also in the Alföld, where the various MN groups show overlapping δ^15^N and δ^13^C values, while the late Esztár group differs by lower δ^15^N values (Fig. 3).

While the Vinča had a demographic and cultural impact on southern TLBK, the intruding Sopot groups rather influenced the formation of the Lengyel cultural group^13,15^. But during the LN, both the Lengyel cultural circle in Transdanubia and (even more importantly) the Tisza culture in the Alföld show an increase in δ^15^N values compared to the MN (Fig. 3), related to the above-mentioned intensification of agricultural practices. One would expect similar dietary habits between the Vinča/Sopot groups and the Lengyel culture, because of their role in shaping the Lengyel culture. However, human Lengyel δ^15^N and δ^13^C values mostly overlap with those of the TLBK, although the few sites providing enough baseline samples revealed a protein-rich diet during the LN. In contrast to the Lengyel group, the elevated δ^15^N values from the Tisza sample are associated with particularly low δ^13^C values, reaching similar levels as for the Sopot group. In this context, it is not excluded that fishing and gathering were again playing an important role for the Tisza communities^28^.

The observed dietary difference compared to Transdanubia may as well be related to the emergence of tell mounds and the increased diversity of land-use activities in the LN Alföld^48,49^. Moreover, the formation of the Tisza culture resulted from the cultural interaction between Szakálhát and late local ALBK groups^16^, but, just as stressed above for the Lengyel group, the diet of these LN individuals notably differs from these two MN samples (Fig. 3). This indicates again that despite a certain degree of continuity at the material culture level, the development of subsistence strategies remained dynamic and specific to each cultural group. The increased socio-political complexity of LN groups further contributes to explain such dietary differences. Previous strontium and oxygen isotope studies further stressed a reduced mobility during the LN^43,50,51^, which may be put into perspective with the intensification of agricultural practices and the increasing use of manure.

### Environmental and climatic diversity

The outlined patterns suggest some essential differences in dietary habits and subsistence strategies not only over time but also between contemporaneous cultural groups. When comparing the results at the cultural group level, it is nevertheless important to note that only seven out of 17 cultural groups are represented by at least five sites. All other cultural groups including the Vinča, Sopot, and Tisza groups are represented by only four sites or less. Thus, there is a risk that the observed isotopic variability might be rather representative for site-specific instead of cultural-specific patterns linked to dietary habits. It is not always possible to assess this directly from the isotopic baseline since animal and plant samples are sometimes scarce. This section discusses a first attempt to test the influence of environmental versus cultural factors on isotope variability.

The cluster subdivision is heavily determined by the geographical location in the Alföld or in Transdanubia (Fig. 5 and S10-S11). Therefore, most contemporaneous cultural groups from these two regions are inevitably associated with different environmental clusters based on this essential geographical difference. Isotopically, it appears that the Alföld δ^15^N values are quasi systematically more elevated than Transdanubian values in both humans and animal samples (figs. S8a and S9a). This seems to be at least partly related to the important role of alluvial and salt affected soils at those sites (tab. S4). However, and despite the small sample size among animals, it is striking that herbivore isotope values are considerably more varied than human values between the environmental clusters, and that the herbivore and human isotope data do not follow the same patterns in terms of isotope variability (fig. S12). This demonstrates that the herbivores are isotopically more sensitive to environmental variability than humans, and in turn, that the human diet is rather standardized and hence reflects culturally-driven habits.

On the diachronic perspective, the restricted distribution of the EN site over three out of nine environmental clusters (fig. S11) may reflect the still constrained settlement spread of early agropastoral communities during the EN^9^. Without ignoring the larger sample size, the fact that MN sites are represented in each environmental cluster may in turn reflect the archaeologically attested expansion in site distribution during the MN^10,13^. The lesser diversity observed for LN and Chalcolithic sites may be related to further archaeologically and isotopically attested shifts in subsistence strategies and site distribution^11,16,17^.

In this context, the three Sopot sites from Hungary show three different soil settings in their catchment area and are distributed over two environmental clusters (tab. S4, fig. S11). The other Transdanubian cultural groups are associated with the same clusters. The two Vinča sites are located very close to each other, both having mainly brown forest soils in their site catchment and being as well on the most frequently represented environmental cluster in Transdanubia among all cultural groups. Tisza sites are predominantly on meadow soils – though one is on salt affected and alluvial soils. They fall in the same clusters than the other cultural groups in the Alföld. Based on this combination of variables, there is thus hardly any environmental pattern that would explain the specific isotope values recorded at those sites. We can therefore conclude that the isotope diversity between archaeological groups indeed reflects culturally-driven dietary habits.

Finally, by testing the potential impact of cultural versus environmental parameters in isotope diversity in Neolithic and Chalcolithic Hungary, this approach demonstrates that the environmental variability not only influences land-use strategies but also the isotopic baseline as mainly reflected by the herbivores. However, and more importantly, it reveals that culturally-driven dietary habits have an even stronger impact on human isotope variability. In future applications of this new approach, it might be worthwhile to test the impact of the research settings on the results by modifying the radius of the site catchment or the environmental parameters included in the analysis. It is not excluded that this approach applied to other or larger research areas would reveal a more important influence of environmental parameters on isotope variability.

## Methods

This study focuses on the results of stable isotope analyses carried out on human and animal bone collagen. This section presents the collected material, the preparation, processing and analysis of bone samples, as well as the statistical and environmental analyses associated with the interpretation of isotope data. The material collected from human and animal skeletal remains, the site abbreviations and lab names as well as the institutions in which the material was stored are listed in table S1. The animals are mainly derived from settlement contexts related to the funeral sites where the humans are coming from, and in most cases, they could be identified as residues of human consumption.

### Human bone collagen

The skeletal remains of 536 human individuals from 53 archaeological sites scattered all over present-day Hungary and parts of Croatia were selected for C and N isotope analyses (Fig. 1; Table 1; tab. S1). There is no ethical issue related to the analysis of these archaeological remains as disclosed in the ethics declaration. Access to the skeletal material was provided by the institutions listed in the Supplementary table S1, sheet “E_Storage institutions” in the controlled framework of the German Research Foundation (DFG), grant number Al 287/10 − 1. Among the analyzed remains, 489 samples from 51 sites met the chronological and quality criteria to be kept and used in this study. In the Alföld, this encompasses samples from the following cultural groups: Körös (6000 − 5500 BCE, *n* = 15), ALBK (5500 − 5000 BCE, *n* = 100), Esztár (5350 − 5000 BCE, *n* = 22), Szakálhát (4900 − 4800 BCE, *n* = 54), Tisza (4900 − 4500 BCE, *n* = 31), and Tiszapolgár (4400 − 4300 BCE, *n* = 8). In Croatia, only the Starčevo (6000 − 5500 BCE, *n* = 14), Baden (3500 − 2700 BCE, *n* = 1), and the Vučedol (3000 − 2400 BCE, *n* = 10) groups are represented. In Transdanubia, the dataset includes samples from the Starčevo (5900 − 5500 BCE, *n* = 31), the TLBK (5500 − 5000 BCE, *n* = 42), the Vinča (5400 − 4500 BCE, *n* = 28), the Sopot (5000 − 4800 BCE, *n* = 25), the Lengyel (4900 − 4400 BCE, *n* = 82), the Balaton-Lasinja (4300 − 3800 BCE, *n* = 13) and the Baden (3500 − 2500 BCE, *n* = 7) cultural groups. Table 1 shows how the sample size could be increased using published data from the literature^4–6,8,52–59^. Rib (*n* = 423) was the preferred sampled material, as their bone remodeling rate best reflects the isotopic composition of the diet in recent years before death. If the ribs were not available or poorly preserved, a skull bone (*n* = 81), long bone (*n* = 46), pelvis bone (*n* = 4), mandibula (*n* = 1), scapula (*n* = 1), or other unspecified bone (*n* = 10) was used instead.

### Animal bone collagen

Within the studied area, 30 sites further provided a total of 217 animal bones (Table 1; tab. S1) to determine C and N stable isotope reference data that characterizes the respective habitats. This sample serves as a proxy to diachronically investigate the ecological parameters of the study area as well as the evolution of husbandry and agropastoral strategies. There is no ethical issue related to the analysis of these archaeological remains as disclosed in the ethics declaration. Access to the skeletal material was provided by the institutions listed in the Supplementary table S1, sheet “E_Storage institutions” in the controlled framework of the German Research Foundation (DFG), grant number Al 287/10 − 1. The represented species are *Bos taurus* (*n* = 63), *Bos primigenius* (*n* = 11), *Bos* unspecified (*n* = 10), *Sus domesticus* (*n* = 34), *Sus scrofa* (*n* = 8), *Sus* unspecified (*n* = 1), *Ovis aries* (*n* = 10), *Capra hircus* (*n* = 1), ovicaprids (*n* = 46), *Ovis/Capra/Sus* uncertain (*n* = 2), *Capreolus capreolus* (*n* = 7), *Cervus elaphus* (*n* = 9), *Lepus europaeus* (*n* = 3), and *Canis familiaris* (*n* = 9). The species of four samples could not be determined. The selected animal bones were primarily long bones or skull fragments.

### Stable isotope analyses

All bone fragments were first superficially ground down, then sawed open and the cancellous bone was removed. Resulting from this preparation, at least 300 mg of sample material was collected per individual. Collagen was isolated using the Longin method^60^, modified according to Müldner and Richards^61^. To determine the relative proportions of C and N, the samples were vaporized and quantified in an element analyser (Vario EL III, Elementar Analysis Systems, Hanau, Germany). The C and N isotopes were measured using an IsoPrimeTM High Performance Stable Isotope Ratio mass spectrometer from GV Instruments, Manchester. Two weights were taken for each sample in order to minimize errors. The mean value was then calculated from both samples. The data was normalized according to Paul and colleagues^62^. In order to exclude diagenesis processes when analyzing bones deposited in the ground, we used the quality criteria developed by Ambrose^63^. The quantitative collagen content, the atomic C/N ratio and the percentage of both elements in the collagen content were used as quality criteria^64^ in order to reject samples that were contaminated or whose isotope ratio was altered.

### Offset and statistical analyses

Measuring the offset between fauna and human isotope values enables to draw conclusions about human diet composition, especially in terms of trophic level. This can however only be done at the site scale and with contemporaneous data. In 34 cases, a site provided contemporaneous humans and fauna were represented by at least two samples each. A sample size of *n* = 2 is not representative for the baseline or for the human diet at the site, but this low limit was chosen to enhance the dataset. The offset in both C and N isotope ratios was calculated from the mean δ^13^C values and mean δ^15^N values of humans compared to those of contemporaneous herbivores and omnivores from the same site.

To test the significance of the observed patterns in terms of diachronic evolution of differences between regions or cultural groups, we performed one-way ANOVA tests and Kruskal-Wallis tests using R-software^65^ and reported the results in the table S2.

### Environmental settings in the study area

The Carpathian Basin at the western edge of the Eurasian Steppe Belt is predominantly influenced by continental climate with some maritime influences resulting in locally and seasonally dry conditions and an annual average rainfall of less than 500 mm in the central plain^66,67^. In the Boreal phase, warmer and drier conditions prevailed, leading to the widespread presence of grassland vegetation^68,69^. During the subsequent Atlantic phase “climatic optimum”, early farming activities and the early Neolithic period in the Carpathian Basin emerged^68^. Large portions of the Great Hungarian Plain are covered by Quaternary gravel, sand and silt^70^ and Upper Pleistocene loess is primarily found along the hilly peripheries of the plain, the Mezőföld region west of the Danube, and the alluvial fans within the basin. Extensive sand deposits are prevalent in the Danube-Tisza Interfluve (DTI) and the Kiskunság region east of the Danube^70^. The eastern part of the Carpathian Basin experienced significant floodplain dynamics and channel reorganization driven by late Pleistocene/Holocene avulsion activity^71–73^. This resulted in a very scattered soil mosaic across the entire Carpathian Basin with a particular abundance of alluvial and meadow soils in the floodplain areas^74^. Lithomorphic soils are abundant in the northern mountain ranges whereas in the loess-covered plains, modern Chernozems predominate and often exhibit significant salt-related soil properties due to the presence of saline groundwater in the central part of the plain^75–77^. In the DTI, sandy soils with localized salt deposits are the dominant soil units^75,77,78^.

### Environmental conditional model

Based on the strong climatic and pedologic variability of the Carpathian Basin, the environmental model integrates large-scale hydroclimatic, topographic, and geomorphological functionalities to evaluate site catchment conditions at the local scale. For this reason, we first establish a monthly weighted model of water accumulation based on topography and precipitation including the entire drainage system of the river Danube upstream of Serbia at a spatial resolution of 150 × 150 m. The model is based on a resampled gridded digital elevation model (DEM) from the SRTM 90 m Digital Elevation Database (v4.1^79^). All statistical and spatial analyses were performed using R software^65^. Using the *whitebox* package^80^, the DEM was cropped to a mask representing the drainage basin of the river Danube (Copernicus, EU-Hydro - River Net User Guide 1.3). All sinks were removed to ensure continuous water accumulation downslope and a raster and vector streamflow system was simulated. From the run-off simulation, the largest connected stream-flow membership and the main stem were extracted. Using the intersection of the main stem and the administrative boundary of Serbia, the upslope area of the river Danube watershed was calculated based on the processed DEM and the intersection point. The study area is cropped to that area that is affected by precipitation occurring across the upslope drainage system of the study area only.

Average monthly precipitation (P) and temperature (T) data for the reference period 1970–2000 was used to estimate rainfall variability across the cropped drainage system at high resolution^81^. P and T gridded data was cropped to that watershed, re-projected, and resampled to the resolution of the DEM. Monthly mean values for P and T were processed for both the entire watershed and for the Carpathian Basin only. We use a combination of the *Rsagacmd*^82^ and the *terra*^83^ packages with the *Rho 8* flow accumulation top down algorithm^84^ from the hydrology tool to estimate the weighted water accumulation as a function of the upslope gradient and the monthly rainfall variability. We equally processed the T average values and cropped the monthly subsets to the extent of the Carpathian Basin core area. P, T, and flow accumulation (ACC) were rescaled using a min-max function.

From the P and T subsets of the Carpathian Basin we created a set of conditional models (CON) to estimate areas that are affected by dry conditions during high T and low P values, humid conditions during high P and low T values and moderate conditions during average climatic variability across each month. We used quantiles as thresholds to differentiate into probabilities below 25%, 25–50%, 50–75% and above 75% of the gridded data range. CON follows the rationale in table S5; the result can be visualized using monthly gridded plots (see repository to this article^19^). The results from monthly CON were then used to create a second weighted conditional model (wCON) that integrates the normalized ACC as a factor to estimate zones of drought vulnerability or increased water availability across the study area (wCON drought index, Fig. 6). We calculated the quartiles of the flow accumulation model (QA) and the conditional model (QC) and applied the following conditions for each month (tab. S6).

The output of wCON integrates regional feedback to supraregional hydroclimatic conditions and the resulting drought/wetness index for the study area (Fig. 6). These two parameters were chosen to best represent monthly variability of dry/humid conditions in the Carpathian Basin. To evaluate the isotopic data from the sample site catchment with a 3000 m radius around each site, we ran cluster analyses on gridded data cropped to spatial buffers of each site. We selected the monthly conditional drought index (wCON, Fig. 6) as well as high-resolution soil dataset^24^ (Fig. 7) that provides information about fertility and potential land-use. The data comes in vector format and covers nine different soil types, depending on morphological conditions (with ID): 10 = skeletal soil, 20 = sand soil, 30 = lithomorphic soil, 40 = brown forest soil, 50 = Chernozem, 60 = salt affected soil, 70 = meadow soil, 80 = peat soil, and 90 = alluvial soil. All raster data within the spatial buffers were transformed into data matrices and *K-means* clustering analyses were performed using variable numbers of clusters (*k*) and the package *stats*^65^ (Fig. 5).

Due to large data variability, we preprocessed the matrices using a Principal Component Analysis (PCA) prior to clustering. We chose values with 99% to be representative for the variance. Using gap statistics and the *silhouette* method from a combination of the R-packages *factoextra*^85^ and *cluster*^86^, we directly assessed the quality of the cluster criteria for each site (*silhouette* method) as well as statistically compared the cluster structure against random distribution using a maximum number of *k* = 30 (gap statistics^87^). We found that k = 9 provides a suitable number for comparison clusters within a 3000 m catchment around each site.

## Electronic supplementary material

Below is the link to the electronic supplementary material.

Supplementary Material 1

Supplementary Material 2

Supplementary Material 3

Supplementary Material 4

Supplementary Material 5

Supplementary Material 6

Supplementary Material 7

Supplementary Material 8

Supplementary Material 9

Supplementary Material 10

Supplementary Material 11

Supplementary Material 12

Supplementary Material 13

Supplementary Material 14

Supplementary Material 15

Supplementary Material 16

Supplementary Material 17

Supplementary Material 18

Supplementary Material 19

Supplementary Material 20

## Data Availability

All data generated and/or analyzed in this study are included in the manuscript. Source Data is available from the supplementary files and from the repository to this article (doi: 10.5281/zenodo.14206253). Material requests should be addressed to: margaux.depaermentier@if.vu.lt; michael.kempf@unibas.ch; Kurt.Alt@dp-uni.ac.at .A commented R code to reproduce the environmental systems analysis, conditional models, and cluster analysis is available from this repository: 10.5281/zenodo.14206253 (During review process, the model code is submitted as additional data file).
