# Supplementary material for: Cultural diversity shaped neolithic subsistence in the Carpathian Basin
Source: Sci Rep. 2025 Feb 4;15:4281. doi: 10.1038/s41598-025-88541-z (PMC11794553; doi:10.1038/s41598-025-88541-z)
Supplement: Supplementary file 1 — Supplementary Material 1 [file 41598_2025_88541_MOESM1_ESM.pdf]

Supplementary Information to: Cultural diversity shaped Neolithic subsistence in the Carpathian Basin

Depaermentier, MLC*[1+], Kempf, M*[2+], Bánffy E[3,4], Alt KW[5,6]

[1]Faculty of History, Vilnius University, Universiteto g. 7, 01513 Vilnius, Lithuania.

[2]Quaternary Geology, Department of Environmental Sciences, University of Basel, Bernoullistrasse 32, 4056 Basel, Switzerland.

[3]Institute of Archaeology, HUN-REN Research Centre for the Humanities, Budapest, Hungary.

[4]German Archaeological Institute, Romano-Germanic Commission, Research Unit Budapest, Hungary

[5]Department of Natural and Cultural Human History, Danube Private University, Krems, Austria.

[6]Institute for Prehistory and Archaeological Science, Basel University, Basel, Switzerland.

MLCD Orcid: 0000-0002-1801-3358. margaux.depaermentier@if.vu.lt
MK ORCID: 0000-0002-9474-4670, michael.kempf@unibas.ch
EB ORCID: 0000-0001-5156-826X, banffy.eszter@abtk.hu
KWA ORCID: 0000-0001-6938-643X, Kurt.Alt@dp-uni.ac.at

Corresponding authors
*margaux.depaermentier@if.vu.lt
*michael.kempf@unibas.ch

+ These authors contributed equally to the paper.

Supplementary texts

Supplementary text S1: Archaeological background

The Carpathian Basin is divided into two major environmental and climatic zones by the so-called Central European Balkan Agro-Ecological Barrier (CEBAEB), with a warmer and drier climate in the South and a more Atlantic-influenced climate in the North-West[1,2]. This barrier is also considered a contact zone between local cultural groups from both regions[3]. The spread of the early agricultural food production system was restricted to the southern part during the Early Neolithic (EN, c. 6000-5500 BCE), because this system was still based on Mediterranean species (i.e., mainly sheep and goats) and traditions[4–7]. The EN agropastoral groups coming

from the Northern Balkans split into two branches in the southern Carpathian Basin, the Körös culture in the East (Alföld) and the Starčevo culture in the West (Transdanubia), with increasing differences in settlement choices, adaptation and coping strategies in the northern margins[8–10].

Archaeozoological records show that fishing and hunting still played a considerable role in the EN subsistence strategies, while domesticates were dominated by sheep and goats – although the proportion of cattle, that could adopt harsher winters, already started to increase and helped the EN communities with dairy products from the beginning[4,5,9,11–14]. Many traces hint on an overlapping period between the end of the late Mesolithic and the start of the early farming settlement in Transdanubia, including shared geographic regions and overlapping absolute chronological dates for the settlement, e.g., in south-eastern Transdanubia with the Regöly and Alsónyék sites[15–17], or in the Balaton region with terminal Mesolithic and late Starcevo-formative LBK settlement[18,19].

However, it is only during the Middle Neolithic (MN, c. 5500-5000/4800 BCE) – and especially starting from the Balaton area in mid-Transdanubia with the Linearbandkeramik (LBK, 5500-5000 BCE) group – that the Neolithic way of life as known in other parts of Europe actually developed[20,21]. From c. 5350 BCE onward, the settlement and agricultural strategies changed, showing a geographically wider and environmentally more diverse distribution pattern, including settlements on loess geologies and Chernozem soils as well as an expansion to the north, beyond the CEBAEB[7,10,22,23]. This was also accompanied by new agricultural techniques and crops, and by a shift towards husbandry strategies dominated by cattle (which was better adapted to the environmental conditions of the Carpathian Basin – especially in the alluvial plains – than the Mediterranean species such as sheep and goats), with a lesser important role of fishing and hunting[22,24]. The expansion to the north, in particular along the Danube and to the east of the Tisza, was clearly connected with the exchange of raw material for flint production

(controlled by local foragers in the mountains), and probably of domesticates and of know-how regarding food production[25]. Long-lasting contacts might also have been related to the need of gaining information about and access to regions rich in salt sources[26]. The genetic ancestry in the EN Carpathian Basin moreover reflects the typical Anatolian descent, characterized by Neolithic package haplogroups with a minimal signal of local ancestry, which, however, increases over time and expanded towards the northwest in later Neolithic centuries[27,28].

Another essential aspect of the MN is the repeated northern Balkan input. At its earliest phase, the Vinča cultural group distributed in southern Transdanubia between 5350 and 5000 BCE[21,29]. The next impact from the Balkans was the intrusion of the Sopot culture (5000-4800 BCE) to Transdanubia, characterized by people with distinct genotypes (with considerable part of Mesolithic admixture in their ancestry) and phenotypes (visible in the osteological remains) as well as specific funeral practices (including diverging body position compared to the contemporaneous TLBK groups[16]. Their settlements were sometimes located close to but separated from the LBK sites and exhibit completely different patterns, also in terms of subsistence strategies[24]. In the east, the Alföld receives similar impacts from the Northern Balkans, which supported the formation of cultural groups such as the Szakálhát (c. 4900-4800 cal BCE). In its northern distribution area, the LBK split into local subgroups such as the Szilmeg, Esztár, Tiszadob and Bükk groups (c. 5350-5000 BCE), to a significant part due to the increasing (also genetically attested) contacts with local foragers[27,30].

In Transdanubia, both the Vinča and Sopot groups played a considerable role in the development of the Late Neolithic (LN) Lengyel group (c. 4900-4400 BCE), which had a vast geographical expansion and can itself be divided into several subgroups based on regional diversity[31–35]. Their agricultural system was more heavily based on cattle herding compared to previous cultures[36] and their settlement distribution changed again, expanding more to brown

forest and skeletal soils as well as to more elevated areas, nevertheless close to water[10,32]. The Lengyel culture had contacts with the Tisza cultural group (ca. 4900-4500 BCE) in the Alföld. The latter was characterized by a dense settlement activity in form of tell mounds[37–39], an increase in political complexity, hierarchy, and social disparities[22], an intensification in the agricultural, foraging, and husbandry practices[40] – which was also shown by the increasing role of manuring[41] – as well as by a restricted mobility[42–44]. The geographical expansion of these two LN cultural groups and the intensification of their agriculture might be related to the optimal climatic conditions of the Atlantic[45].

During the Chalcolithic (c. 4500/4400-2700. BCE) in the Alföld, new areas were selected for the settlements, a great diversity of scattered villages replaced the large LN tells, and the agriculture seemed to evolve to a mobile cattle herding[46,47] – although hardly any evidence for transhumance, mobility, or an important role of diary could be attested from isotope research so far[41,42,48]. The changes between the LN and the Chalcolithic may highlight a Chalcolithic society that was better integrated into more diverse parts of the environment compared to the LN[46,48]. In Transdanubia, the Balaton-Lasinja complex (c. 4300-3800 BCE) resulted yet again in another wave of inputs from the northern Balkans, gradually fading northward. The settlement choice and structure resemble the Lengyel inheritance, but the Balaton-Lasinja communities herded cattle and had a more mobile lifestyle[44,49].

Supplementary text S2: Environmental settings

The Carpathian Basin at the western edge of the Eurasian Steppe Belt has undergone significant environmental and socio-cultural changes throughout the Holocene, partly driven by shifts in climatic conditions[10,50]. The region is predominantly influenced by continental climate with some maritime influences[51,52] resulting in locally and seasonally dry conditions and an annual average rainfall of less than 500 mm in the central plain[53,54]. Regional diversification

during the Holocene was further intensified by arid winds, resulting in the reduction of vegetation cover to steppe or forest-steppe mosaics and led and the accumulation of dust and sand deposits[55–60].

In the Boreal phase, warmer and drier conditions prevailed, leading to the widespread presence of grassland vegetation[57,61,62]. During the subsequent Atlantic phase “climatic optimum”, early farming activities and the early Neolithic period in the Carpathian Basin emerged[62–64].

Large portions of the Great Hungarian Plain (Alföld) are covered by Quaternary sediments comprising gravel, sand, silt, and clay [65]. The Upper Pleistocene loess is primarily found along the hilly peripheries of the plain, the Mezőföld region west of the Danube, and the alluvial fans within the basin. These deposits can frequently alternate with layers of sand and paleosols formed during warmer and wetter interglacial periods. Notably, extensive sand deposits are prevalent in the Danube-Tisza Interfluve and the Kiskunság region east of the Danube[65]. The Transdanubian Range (Dunántúli-Középhegység) extends from the Keszthely mountains to the Pilis-Visegrád mountains. It primarily comprises Triassic formations, with sporadic outcrops of Palaeozoic patches in the Velence mountains and extensive Mesozoic formations in the Bakony and Gerecse mountains. The eastern part (Bükk mountains) primarily consists of thick Middle-Upper Triassic shallow marine carbonates and deep marine sediments, which underwent significant metamorphic folding, uplift, and denudation during the Cretaceous and subsequent periods.

In contrast, Pleistocene-Holocene aeolian sandy sediments are predominantly found in the Danube-Tisza Interfluve (Kiskunság, DTI), and the Nyírség region. The floodplains are characterized by Holocene fluvial deposits[65] and significant floodplain dynamics and channel reorganization during the late Pleistocene and the Holocene[66–68]. The extensive alluvial plain along the Danube River is delimited to the east by an elevated plateau-like palaeo-alluvial fan. This fan was later incised during flooding events and avulsion processes, leading to the shifting

of alluvial deposits. The river Tisza, a major tributary of the Danube, plays a crucial role in this hydrological system[69] and is characterized by extensive alluvial fans with remnants of palaeochannels. These fluvial deposits primarily consist of fine-grained material such as clay, silt, and sand[53,66,67,69] and have been frequently remobilized across the entire floodplain due to channel shifting and avulsion events[53,69].

Local soil development and quality are significantly influenced by fluvial deposits. This has resulted in a very fine-grained soil mosaic[70]. Lithomorphic soils are abundant in the northern mountain ranges and on eroded slopes composed mainly of limestone, dolomite, basalt, and andesite. In the loess-covered plains, modern Chernozems predominate and often exhibit significant salt-related soil properties due to the presence of saline groundwater in the central part of the plain[61,71–73]. In the interfluve, sandy soils with localized salt deposits are the dominant pedological units[71,73,74].

Supplementary text S3: Detailed overview of dietary habits and subsistence strategies

Overall, the C isotope composition of human and animal bones shows a typically C_3-plants-based diet for all regions and periods. However, three animals and three humans exhibit $\delta^{13}C$ values above -18.00‰, which is considered evidence for a diet including C_4 plants[75,76], especially at places located far away from the sea and any marine food, such as Hungary[77]. In this case, it is noteworthy that except for juvenile human BENA06 ($\delta^{15}N$: 12.34‰), the $\delta^{15}N$ values of these individuals and animals are not particularly high, reducing the probability that such elevated $\delta^{13}C$ values may be related to aridity, salty soil, manuring, or marine fish consumption and hence pointing more towards C_4 plants. However, there is no wild C_4 plants attested in this area for this period and recent studies revealed a much later arrival of millet in this region based on paired radiocarbon and stable isotope data on both human osteological remains and millet grains[76,78,79]. Since no radiocarbon date is available, the affiliation of these

samples to the Neolithic or Chalcolithic periods should be considered with caution. In addition, most animals showing $\delta^{13}C$ values above or close to -18.00‰ originate from the southern part of the LBK Balatonszarszo-Kis-erdei-dűlő site. Their elevated $\delta^{13}C$ values were interpreted by A. Whittle and colleagues[80] as an evidence for a diversification in husbandry strategies in the later LBK phase.

On the other hand, 19 animals (when omitting freshwater fish samples) have $\delta^{13}C$ values below -22.00‰, which can be considered a threshold for a diet based on forested environments[81,82]. Expectantly, these are mostly *Cervus elaphus* but also include the *Sus scrofa* and *domesticus*, the *Lepus*, rodentia, and *Canis familiaris*. As for the humans, among the eight individuals having $\delta^{13}C$ values below -22.00‰, four are from a Körös site at Tiszaszőlős-Domaháza (TIDO), located close to the river. Their low $\delta^{13}C$ values combined with high $\delta^{15}N$ values (above 12.00‰) suggest the consumption of freshwater fish at this site during the Körös period. This is supported by the particularly large offset in $\delta^{15}N$ values between human and herbivores/omnivores at this site (>5‰) as well as by archaeozoological records that show a low fraction of domesticated animal at TIDO [83]. The other three humans with low $\delta^{13}C$ values are coming from the Lengyel site at Alsónyék – Bátaszák-Lajvér (BAL), and one comes from the Szakálhát site at Cegléd Ipari park (CGIP). In these cases, an important part of the diet might have been issued from forested areas. At all other sites from this and previous studies, the food predominantly came from opened environments or fields.

Moreover, the results of C and N isotope analyses follow the expectations related to the position of each animal species in the food chain. When considering only the main species (and hence omitting fish, horses, hares, and rodents), the cervidae (in particular the *Capreolus capreolus*) overall show the lowest $\delta^{13}C$ values, followed by the suids (especially *Sus scrofa*) and the ovicaprids, while the *Canis* and *Bos* species have the highest $\delta^{13}C$ values. Regarding N, the cervidae show yet again the lowest isotope ratios, followed by the ovicaprids and the *Bos*

species. Among the latter, the *Bos primigenius* has overall slightly overlapping but higher $\delta^{15}N$ values compared to *Bos taurus*. The suids have the second highest and the dogs expectantly the highest $\delta^{15}N$ values. This suggests that the cervidae and to some extent the *Sus* species got at least parts of their diet from forested areas, while the *Bos* species spent most of their time in open landscapes. The difference in $\delta^{15}N$ values between *Sus scrofa* and *Sus domesticus* further suggest that the latter may have had access to food residues from the human's diet.

At half of the sites, i.e. mostly at EN and LN sites, the offset in $\delta^{15}N$ values between contemporaneous humans and herbivores/omnivores represents a normal trophic level shift between human and fauna, reflecting the important role of animal proteins in human diet during the EN and the LN. At the other half of the sites, predominantly dated to the MN, the offset in $\delta^{15}N$ values is below 3‰ and suggests a less important part of animal proteins in the human diet during the MN. At one site, the above-mentioned TIDO, the offset in $\delta^{15}N$ values is slightly above 5‰, which suggests a higher amount of animal protein in the human diet compared to the other sites – or the input of proteins from higher trophic levels, such as freshwater fish. The offset in $\delta^{13}C$ values between contemporaneous humans and fauna samples overall reveals that the EN sites – especially from the Körös group – often exhibit humans with lower $\delta^{13}C$ values than animals. This suggests that earliest farmers in this area were possibly including more food from forested or freshwater ecosystems in their diet compared to the later groups. This assumption is supported by archaeozoological analyses revealing that fishing and hunting were important at many EN/Körös sites[4] and played overall a constant role in human diet as complement to the meat and dairy products provided by the domesticates[5,84].

Supplementary figures

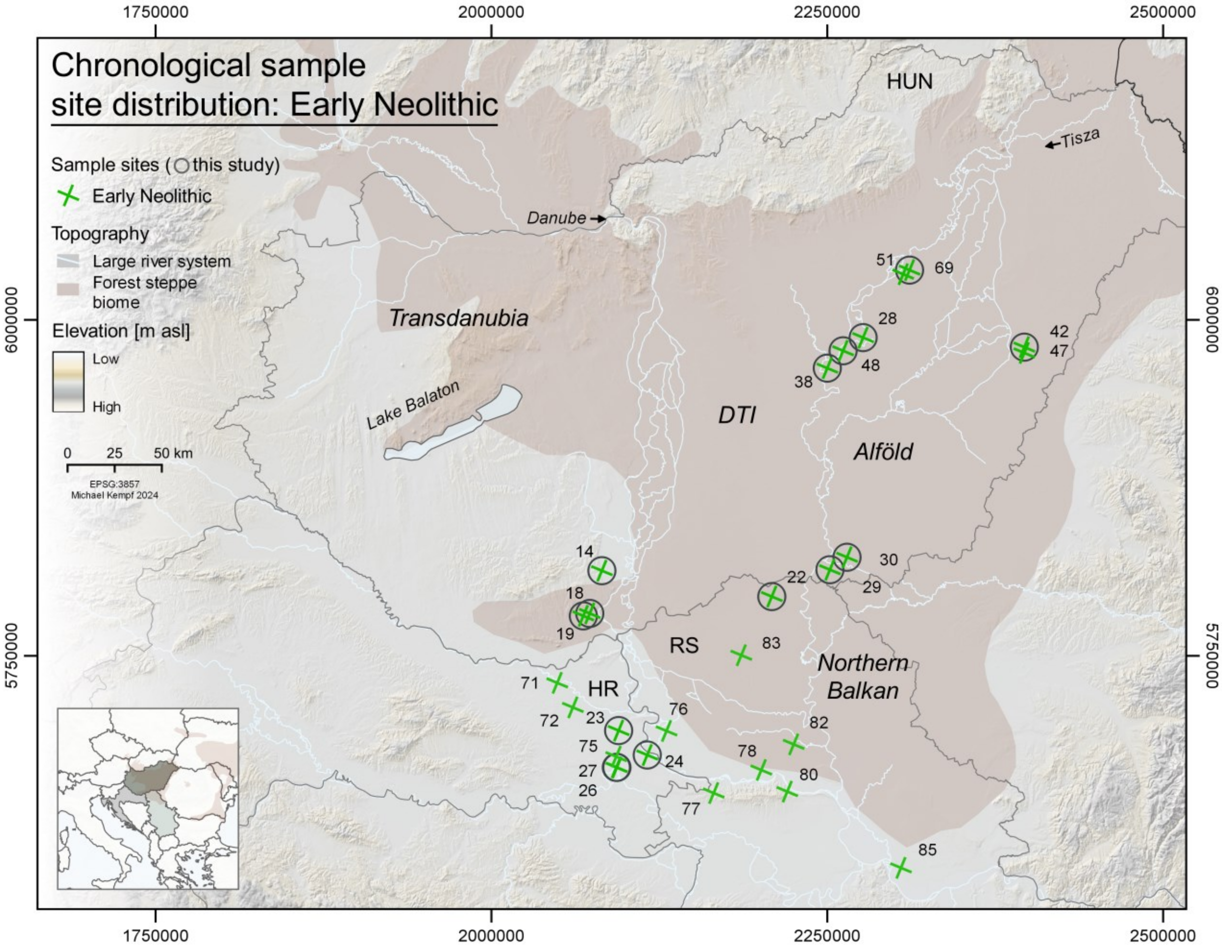

Supplementary fig. S1| Geographical distribution of the studied sites from the Early Neolithic (EN). Site IDs and reference to the data from the literature are listed in table S1. DTI = Danube-Tisza Interfluve; Modern country borders: HUN = Hungary; HR = Croatia; RS = Serbia. This map is produced using QGIS 3.10.12 (QGIS Geographic Information System. QGIS Association. http://www.qgis.org (2024)).

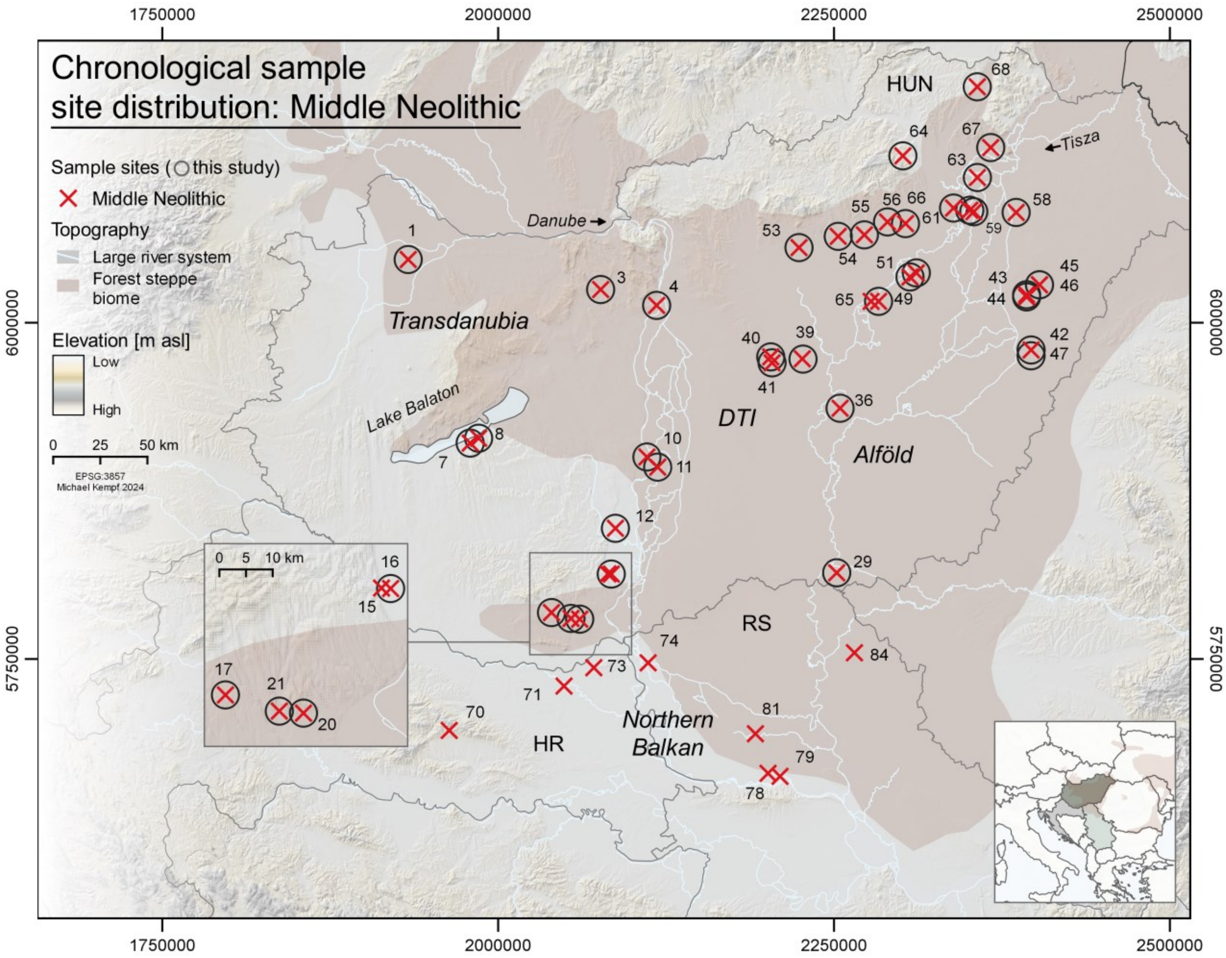

Supplementary fig. S2| Geographical distribution of the studied sites from the Middle Neolithic (MN). Site IDs and reference to the data from the literature are listed in table S1. DTI = Danube-Tisza Interfluve; Modern country borders: HUN = Hungary; HR = Croatia; RS = Serbia. This map is produced using QGIS 3.10.12 (QGIS Geographic Information System. QGIS Association. http://www.qgis.org (2024)).

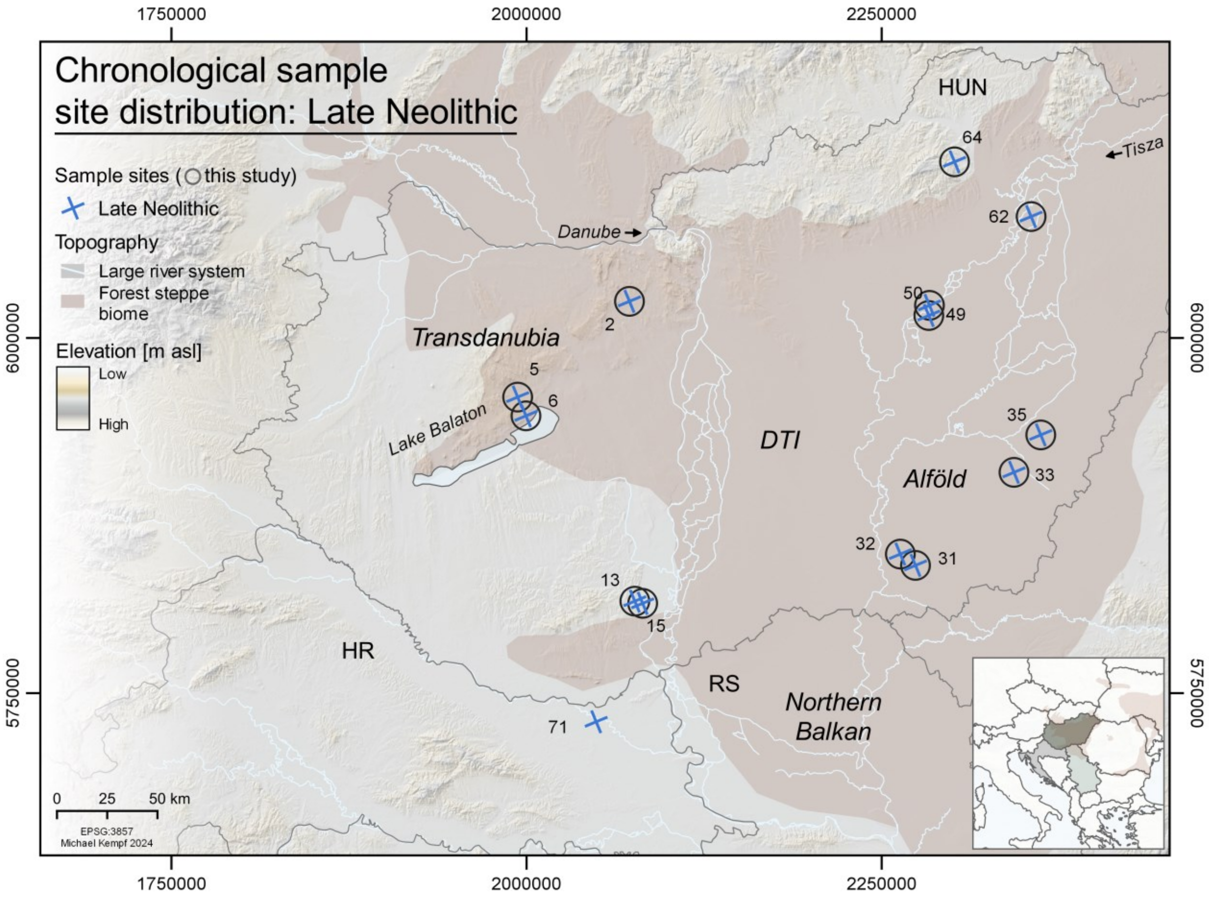

Supplementary fig. S3| Geographical distribution of the studied sites from the Late Neolithic (LN). Site IDs and reference to the data from the literature are listed in table S1. DTI = Danube-Tisza Interfluve; Modern country borders: HUN = Hungary; HR = Croatia; RS = Serbia. This map is produced using QGIS 3.10.12 (QGIS Geographic Information System. QGIS Association. http://www.qgis.org (2024)).

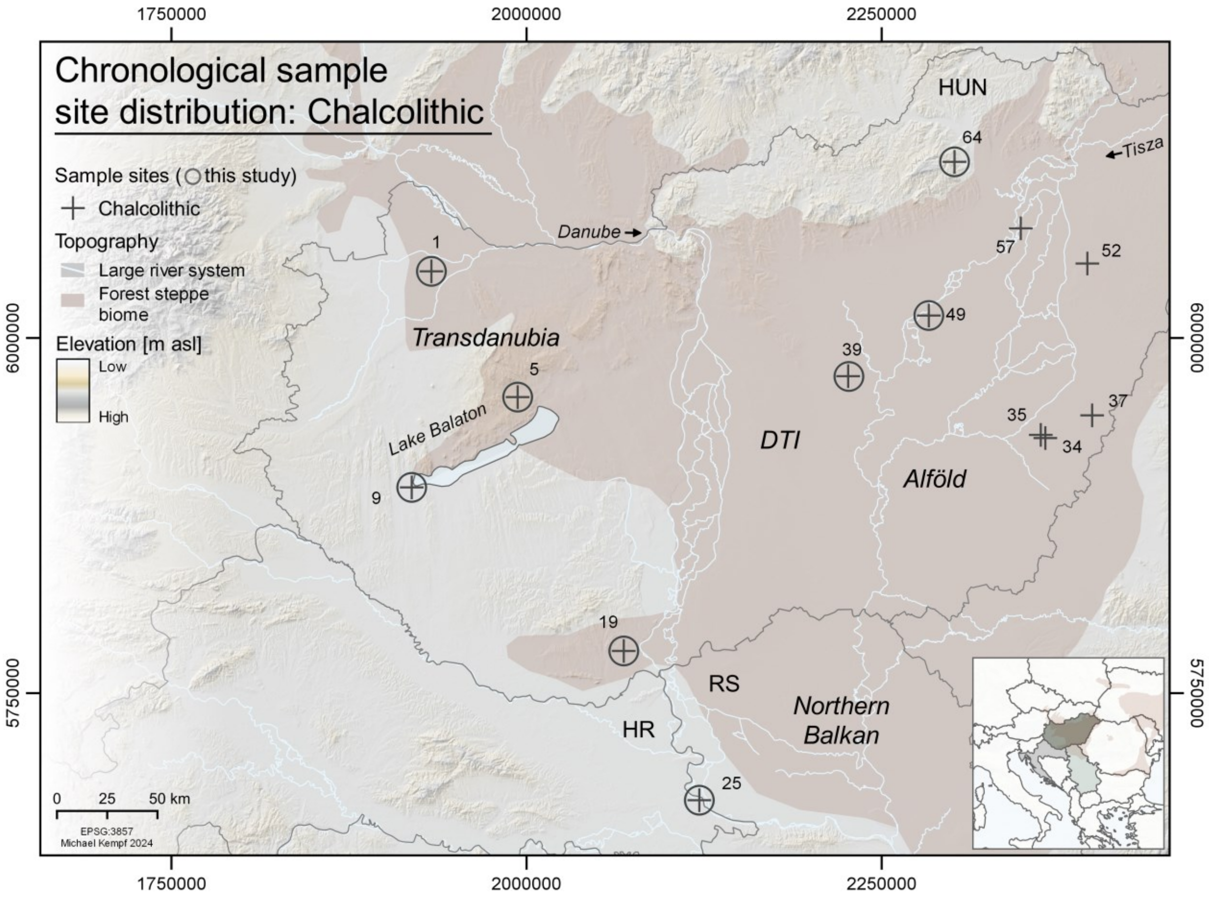

Supplementary fig. S4| Geographical distribution of the studied sites from the Chalcolithic. Site IDs and reference to the data from the literature are listed in table S1. DTI = Danube-Tisza Interfluve; Modern country borders: HUN = Hungary; HR = Croatia; RS = Serbia. This map is produced using QGIS 3.10.12 (QGIS Geographic Information System. QGIS Association. http://www.qgis.org (2024)).

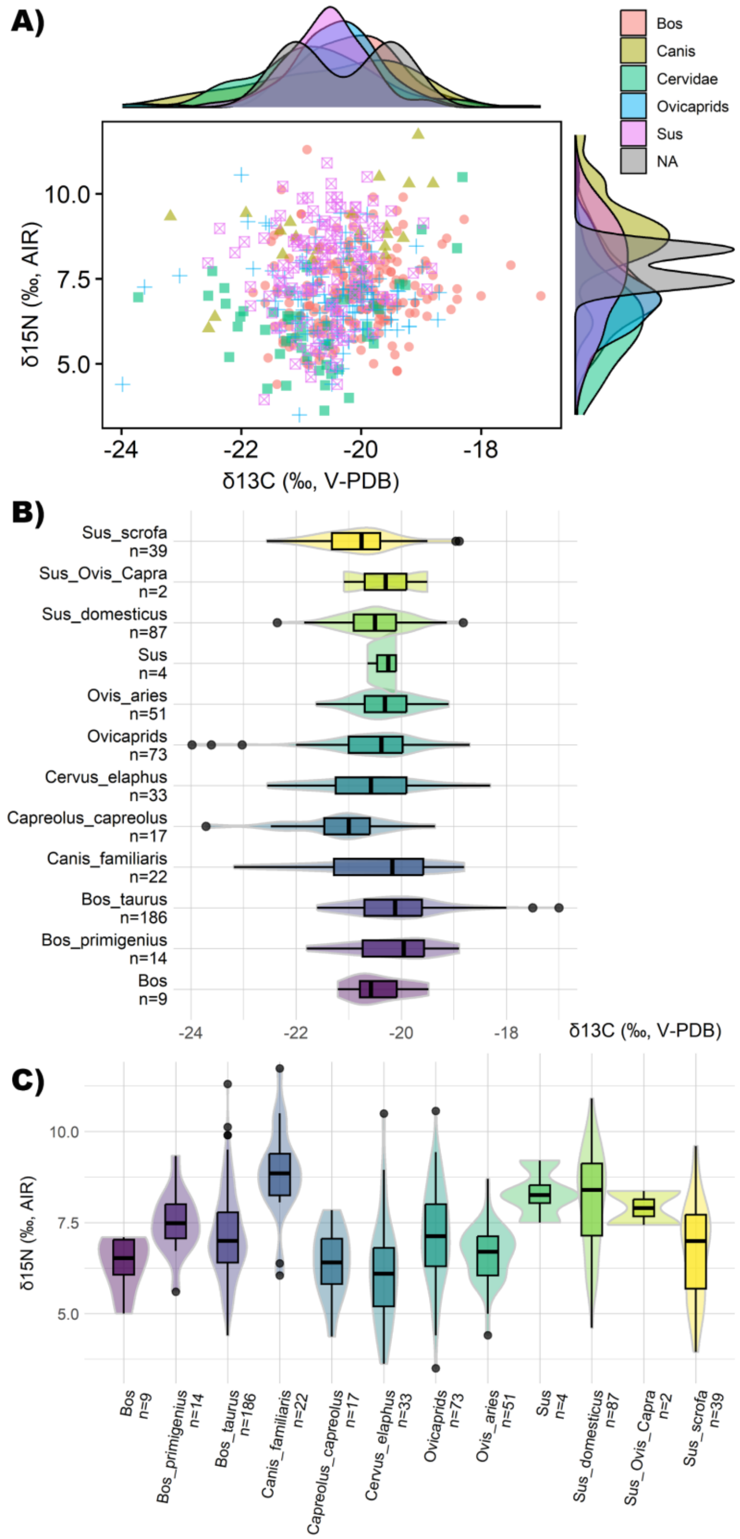

Supplementary fig. S5|Carbon and nitrogen isotope values among the animal species. a) Overview of the $\delta^{13}C$ and $\delta^{15}N$ values of the main animal groups. **b)** Animal $\delta^{13}C$ values among species. **c)** Animal $\delta^{15}N$ values among species.

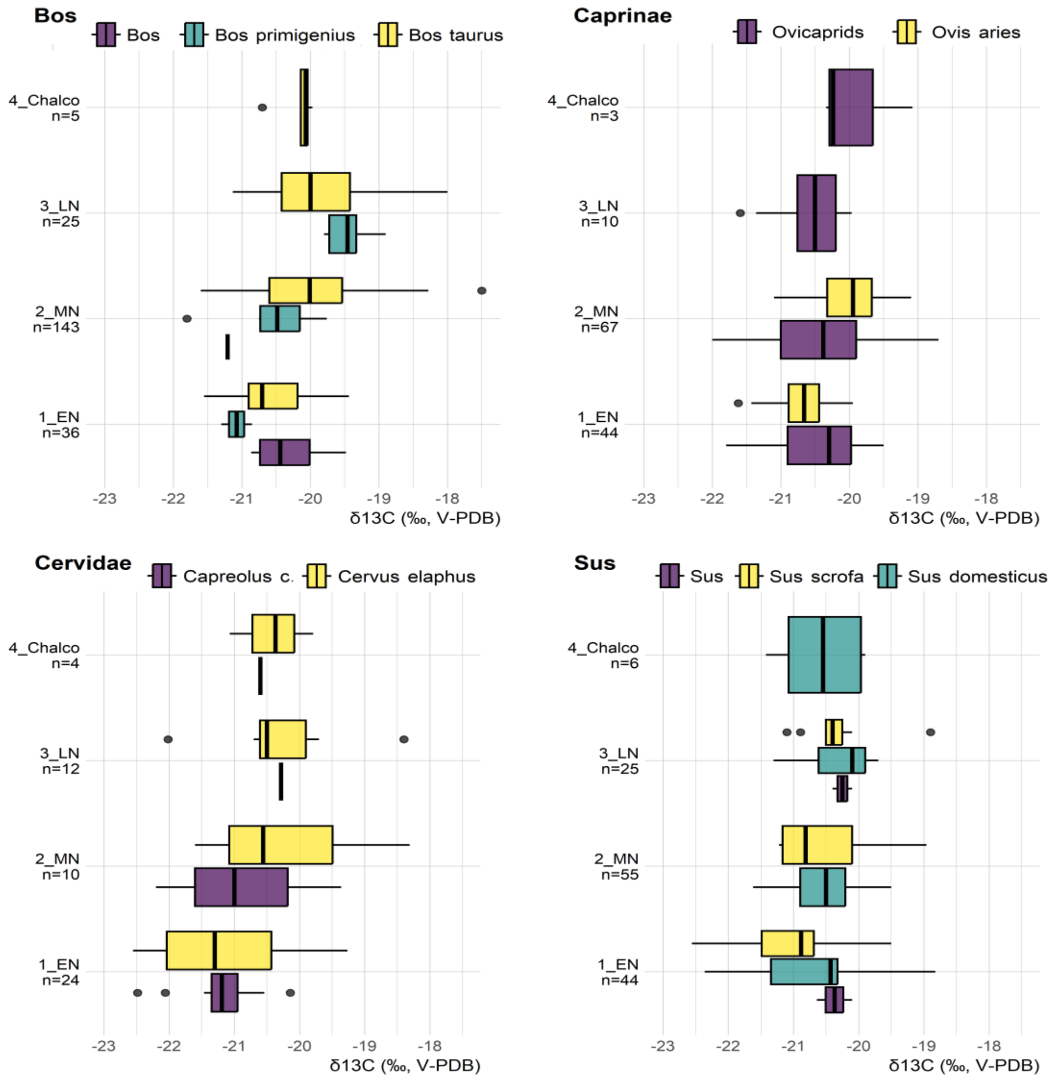

Supplementary fig. S6|Diachronic evolution of the $\delta^{13}C$ values among the main animal species.

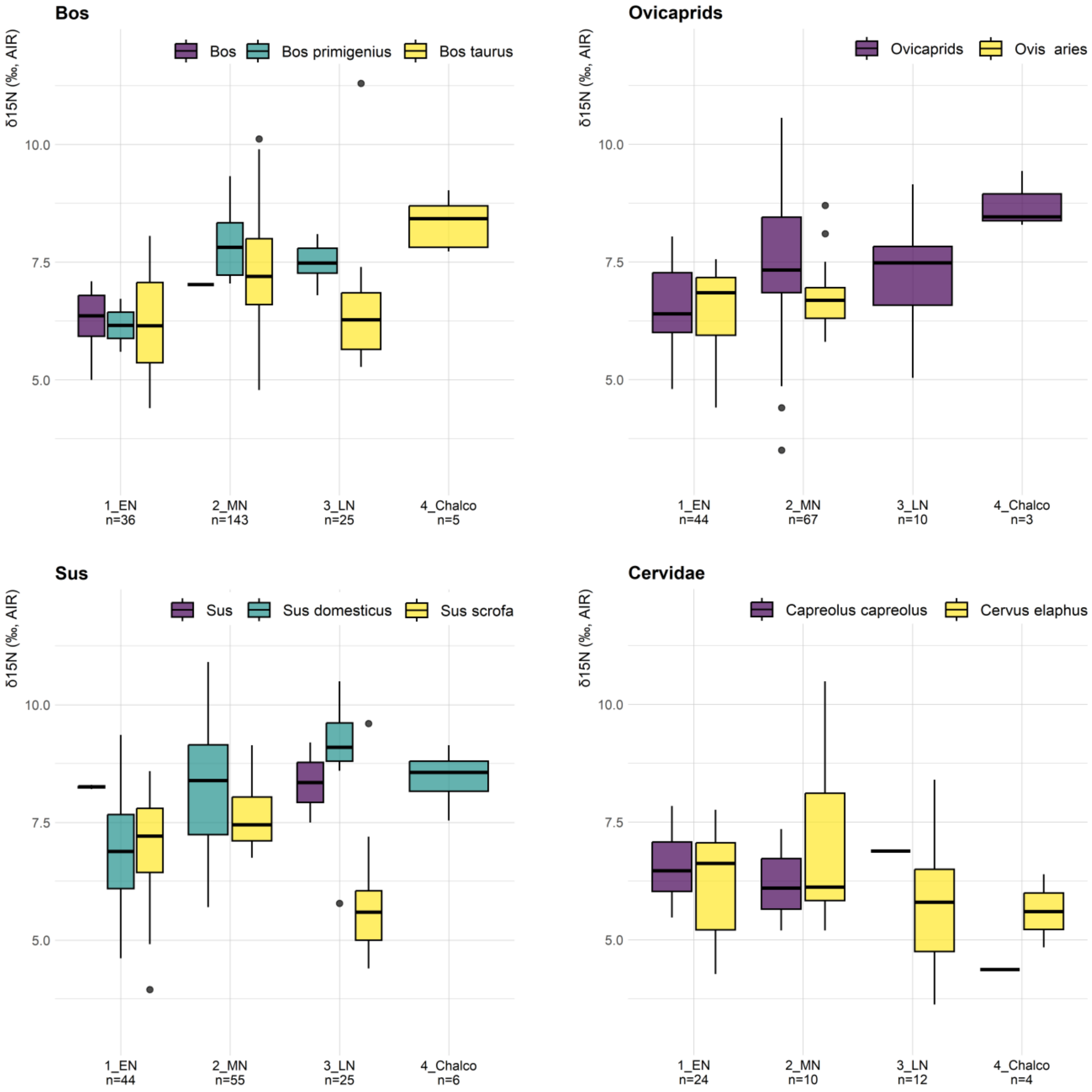

Supplementary fig. S7|Diachronic evolution of the δ^{15}N values among the main animal species.

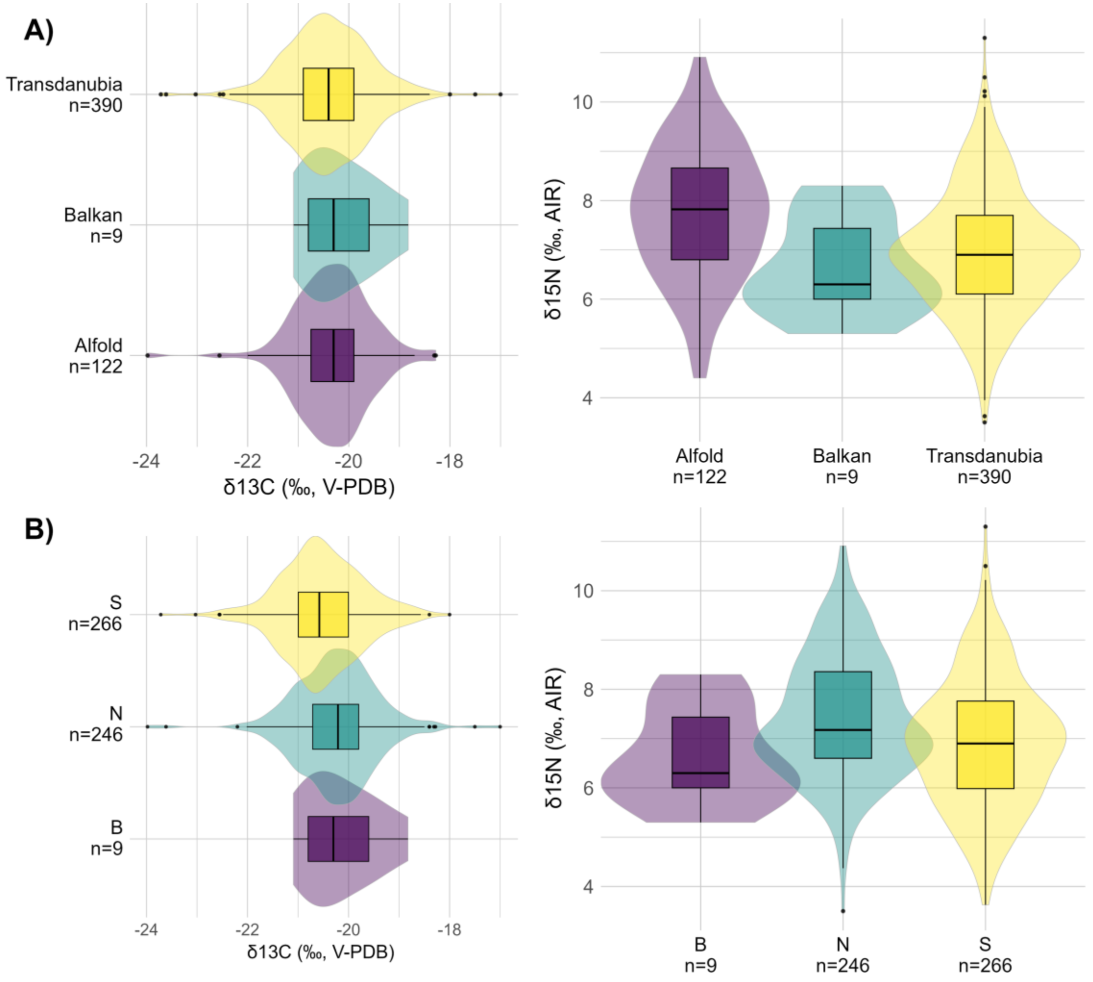

Supplementary fig. S8|Geographical differences in animal δ^{13}C and δ^{15}N values (including the main herbivores and omnivores only). a) Animal δ^{13}C and δ^{15}N values between the main regions. **b)** Animal δ^{13}C and δ^{15}N values between southern (S) and northern (N) Hungary and the northern Balkans (B).

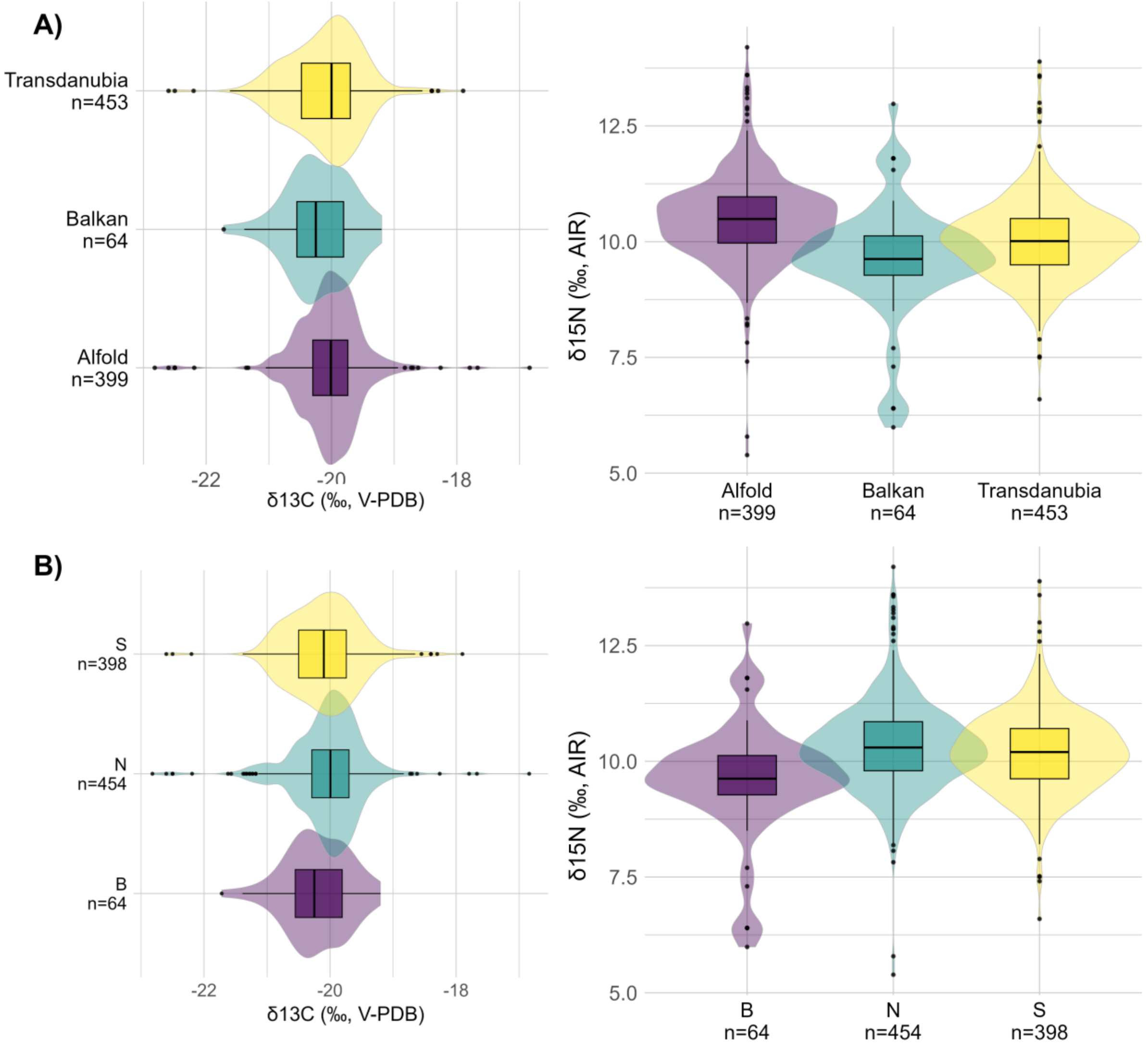

Supplementary fig. S9|Geographical differences in human $\delta^{13}C$ and $\delta^{15}N$ values between.
a) Human $\delta^{13}C$ and $\delta^{15}N$ values between the main regions. **b)** Human $\delta^{13}C$ and $\delta^{15}N$ values between southern (S) and northern (N) Hungary and the northern Balkans (B).

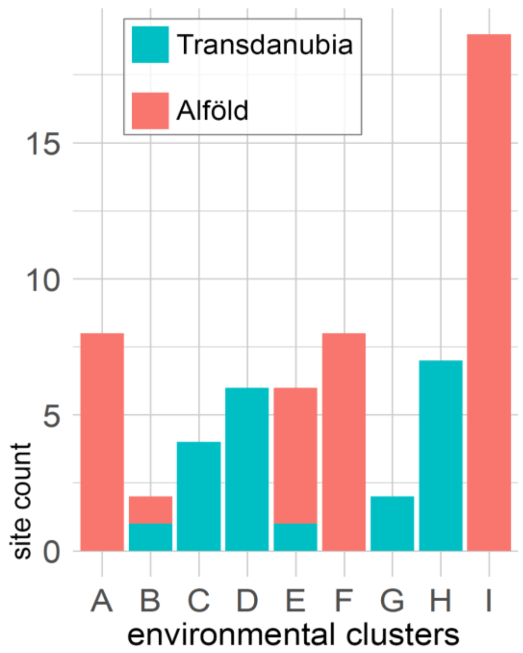

Supplementary fig. S10|Number of sites from the two main regions within the various environmental clusters. Using the nine statistically determined environmental clusters.

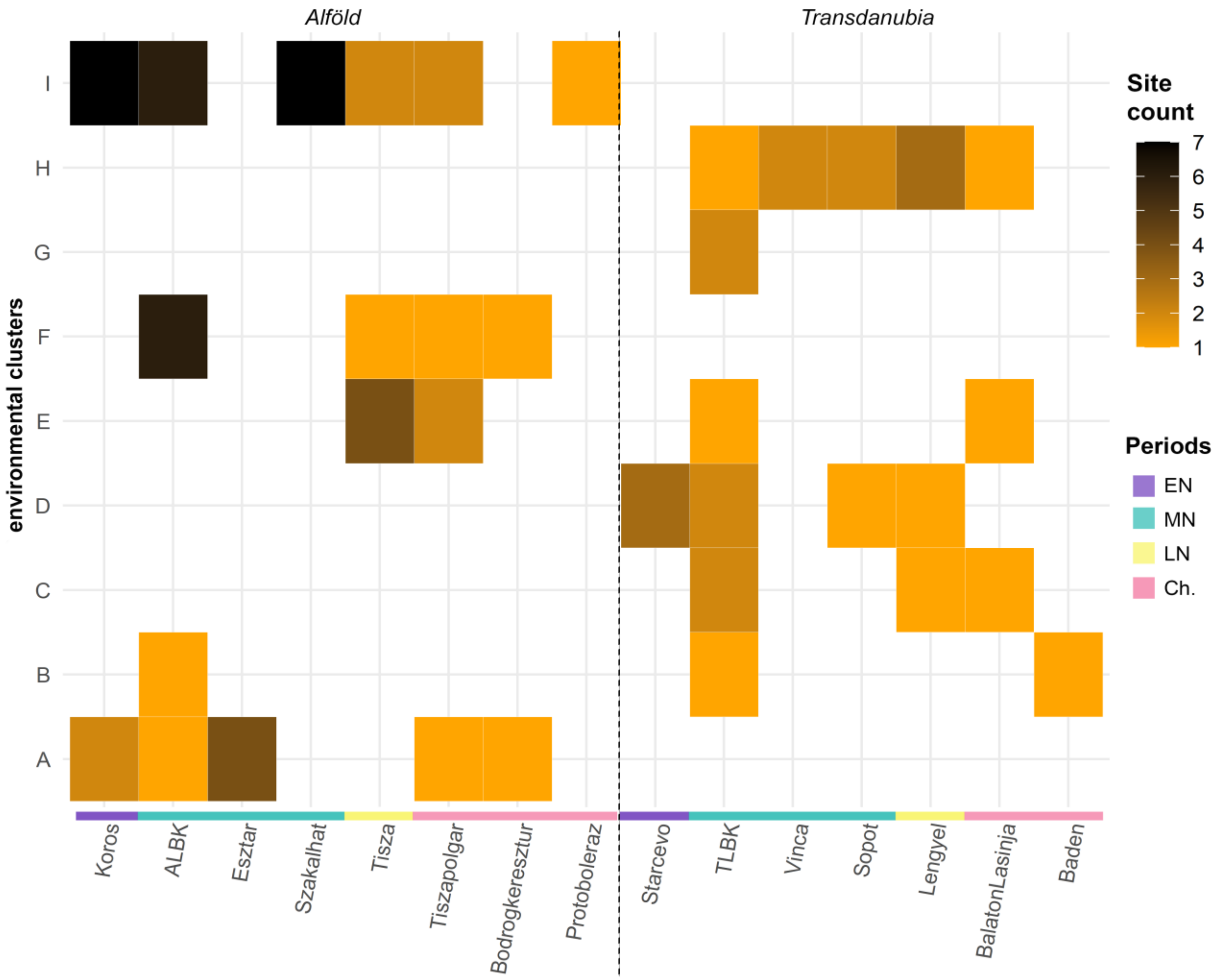

Supplementary fig. S11|Number of sites from the studied cultural groups within the various environmental clusters. Using the nine statistically determined environmental clusters.

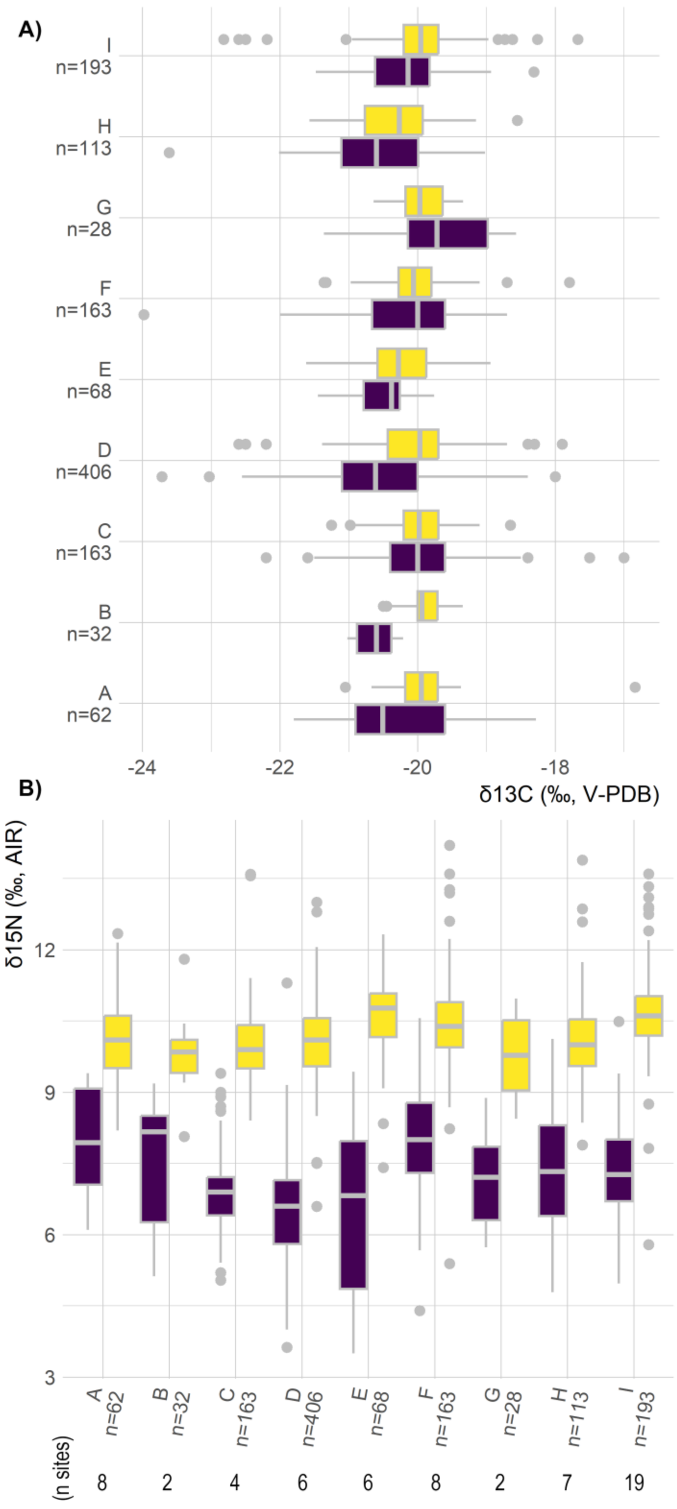

Supplementary fig. S12|Isotope variability of the human (yellow) and herbivore (purple) samples among the various environmental clusters. a) Human and herbivore carbon isotope values over the nine statistically determined environmental clusters. **b)** Human and herbivore nitrogen isotope values over the nine statistically determined environmental clusters.

Supplementary tables

[see Excel file online]

Supplementary table S1|Detailed list of the results and collected data. a) Details of the human isotope data used for this study. **b)** Details of the human isotope data from the samples excluded from this study due to bad quality criteria. **c)** Details of the animal isotope data used for this study. **d)** Details of the animal isotope data from the samples excluded from this study due to bad quality criteria. **e)** List by archaeological site of the storage institution from which we collected samples for isotope analysis. **f)** List of the references for this study and for the published data included in this study.

**test type →**	**one way ANOVA test**				**Kruskal-Wallis test**			
**tested issue ↓**	**Df**	**F value**	**p-value**	**significant?**	**Df**	**chi-squared**	**p-value**	**significant?**
changes in fauna δ13C over time	3	18.520	1.92E-11	yes***	3	54.44	2.41E-11	yes***
changes in fauna δ15N over time	3	11.780	1.77E-07	yes***	3	35.08	1.17E-07	yes***
changes in cervidae δ13C over time	3	3.745	0.0173	yes*	3	52.44	2.41E-11	yes***
changes in cervidae δ15N over time	3	2.058	0.119	no	3	5.03	0.1696	no
differences in fauna δ13C between main regions	2	1.799	0.166	no	2	3.66	0.1608	no
differences in fauna δ15N between main regions	2	14.940	4.85E-07	yes***	2	27.98	8.40E-07	yes***
differences in fauna δ13C between North and South	2	9.809	6.55E-05	yes***	2	20.5	3.55E-05	yes***
differences in fauna δ15N between North and South	2	5.963	0.00275	yes**	2	10.61	0.004965	yes***
changes in human δ13C over time	3	31.460	<2e-16	yes***	3	90.525	< 2.2e-16	yes***
changes in human δ15N over time	3	8.228	2.09E-05	yes***	3	35.17	1.12E-07	yes***
differences in human δ13C between cultural groups	16	10.240	<2e-16	yes***	16	173.94	< 2.2e-16	yes***
differences in human δ15N between cultural groups	16	9.176	<2e-16	yes***	16	181.05	< 2.2e-16	yes***
differences in human δ13C between main regions	2	3.195	0.0414	yes*	2	6.4638	3.95E-02	yes*
differences in human δ15N between main regions	2	38.920	<2e-16	yes***	2	84.776	< 2.2e-16	yes***
differences in human δ13C between North and South	2	3.259	0.0389	yes*	2	11.169	3.76E-03	yes**
differences in human δ15N between North and South	2	15.930	1.59E-07	yes***	2	26.905	1.44E-06	yes***
differences in herbivore δ13C between 9 clusters	8	5.196	3.63E-06	yes***	8	41.07	2.02E-06	yes***
differences in herbivore δ15N between 9 clusters	8	7.920	7.53E-10	yes***	8	51.7	1.92E-08	yes***
differences in human δ13C between 9 clusters	8	3.797	0.000216	yes***	8	34.75	2.97E-05	yes***
differences in human δ15N between 9 clusters	8	9.774	4.85E-13	yes***	8	100.11	< 2.2e-16	yes***
**Chi-square test**	**Df**	**X-squared**	**p-value**	**significant?**				
environmental clusters over main regions (9 clusters)	8	56.047	2.76E-09	yes***				

Signif. codes: 0 '***' 0.001 '**' 0.01 '*' 0.05 or 0.1 or 1 'no'

Supplementary table S2|Results of the statistical tests. Results of the on-way ANOVA tests, the Kruskal-Walli tests, and the Chi-square test applied in this study. Signif. codes: 0 '***' 0.001 '**' 0.01 '*' 0.05 or 0.1 or 1 'no'. *[see also Excel file online].*

[see Excel file online]

Supplementary table S3|Offset data by site. Summary by site of the mean values for human and animal $\delta^{13}C$ and $\delta^{15}N$ values and corresponding offsets in $\delta^{13}C$ and $\delta^{15}N$ values between humans and fauna. This includes only sites where the sample size was at a minimum n=2 for humans and/or animals.

[see Excel file online]

Supplementary table S4|Environmental clusters and soil settings by site. Information, for each site, about the affiliation to statistically determined environmental clusters as well as about dominant soils and the percentage of each main soil type within the catchment area.

Condition	Description	Formula for Vi
Ti > QT[3] and Pi > QP[3]	Both temperature and precipitation are in the upper quartile	Vi = (Ti + Pi) / 2
Ti > QT[3] and Pi < QP[1]	Temperature is in the upper quartile, precipitation is in the lower quartile	Vi = (Ti × 2 + Pi) × (-2)
Ti < QT[1] and Pi < QP[1]	Both temperature and precipitation are in the lower quartile	Vi = (Ti + Pi) × (-1)
Ti < QT[1] and Pi > QP[3]	Temperature is in the lower quartile, precipitation is in the upper quartile	Vi = (Ti + Pi × 2) × 2
Ti > QT[3] and QP[1] ≤ Pi ≤ QP[3]	Temperature is in the upper quartile, precipitation is in the middle quartiles	Vi = (Ti + Pi) × (-1)
Ti < QT[1] and QP[1] ≤ Pi ≤ QP[3]	Temperature is in the lower quartile, precipitation is in the middle quartiles	Vi = (Ti + Pi) × 1.5
QT[1] ≤ Ti ≤ QT[3] and Pi > QP[3]	Temperature is in the middle quartiles, precipitation is in the upper quartile	Vi = Ti + Pi
QT[1] ≤ Ti ≤ QT[3] and Pi < QP[1]	Temperature is in the middle quartiles, precipitation is in the lower quartile	Vi = (Ti + Pi) × (-1.5)
QT[1] ≤ Ti ≤ QT[3] and QP[1] ≤ Pi ≤ QP[3]	Both temperature and precipitation are in the middle quartiles	Vi = (Ti + Pi) / 2
Otherwise	Any other case	Vi = NA

Supplementary table S5|CON statements (V) for precipitation (P) and temperature (T) dependent on quartiles of the data range (QP and QT) for each season.

Condition	Description	Formula for Vi
ACC > QA[3] and CON > QC[3]	Both ACC and CON in the upper quartile	Vi = (ACC \× 3 + CON)
ACC > QA[3] and CON < QC[1]	ACC in upper quartile, CON in lower quartile	Vi = (ACC × 2 - CON)
ACC < QA[1] and CON < QC[1]	Both ACC and CON in the lower quartile	Vi = (CON × -1.5 + ACC)
ACC < QA[1] and CON > QC[3]	ACC in lower quartile, CON in upper quartile	Vi = (CON + ACC)
ACC > QA[3] and QC[1] ≤ CON ≤ QC[3]	ACC in upper quartile, CON in middle quartiles	Vi = (ACC × 0.5 + CON × 0.5)
ACC < QA[1] and QC[1] ≤ CON ≤ QC[3]	ACC in lower quartile, CON in middle quartiles	Vi = (CON × 0.25 + ACC × 0.25)
QA[1] ≤ ACC ≤ QA[3] and CON > QC[3]	ACC in middle quartiles, CON in upper quartile	Vi = (ACC × 2 + CON)
QA[1] ≤ ACC ≤ QA[3] and CON < QC[1]	ACC in middle quartiles, CON in lower quartile	Vi = (CON × -1 + ACC × 2)
QA[1] ≤ ACC ≤ QA[3] and QC[1] ≤ CON ≤ QC[3]	Both ACC and CON in middle quartiles	Vi = mean(ACC + CON)
Otherwise	Any other case	Vi = NA

Supplementary table S6 | wCON statements (V) for flow accumulation (ACC) and the climatic conditional model output (CON) dependent on quartiles of the data range (QA and QC) for each month.

References

1. Sümegi, P. Environmental changes under the neolithization process in Central Europe: before and after. *Anthaeus* **27,** 117–128 (2004).

2. Sümegi, P. Paleogeographical background of the Mesolithic, Early Neolithic settlements in the Carpathian Basin. *Proceedings of the 15th UISPP,* 47–51 (2008).

3. Bánffy, E. & Sümegi, P. The early Neolithic agro-ecological barrier in the Carpathian Basin: a zone for interaction. In *Archaeological, cultural and linguistic heritage: Festschriftfor Erzsébet Jerem in honour of her 70th birthday,* edited by P. Anreiter, E. Bánffy, L. Bartosiewicz, W. Meid & C. Metzner-Nebelsick (Archaeolingua, Budapest, 2012), pp. 57–69.

4. Kovács, Z. E. & Bartosiewicz, L. Early Neolithic animal bones from Ibrány-Nagyerdő, Hungary. In *Neolithization of the Carpathian Basin: Northernmost distribution of the Starčevo/Körös culture,* edited by J. K. Kozłowski & P. Raczky (Polska Akademia Umiejętności, Kraków, 2010), pp. 236–252.

5. Bartosiewic, L. Early Neolithic Fishing in the Middle Tisza Region, Hungary. *Archeofauna* **22,** 133–144 (2013).

6. Ethier, J., Bánffy, E., Vuković, J., Leshtakov, K., Bacvarov, K., Roffet-Salque, M., Evershed, R. P. & Ivanova, M. Earliest expansion of animal husbandry beyond the Mediterranean zone in the sixth millennium BC. *Scientific Reports* **7,** 7146; 10.1038/s41598-017-07427-x (2017).

7. Gulyás, S., Nagy, B., Sümegi, P., Schöll-Barna, G. & Demény, A. Intensified mid-Holocene floods recorded by archeomalacological data and resilience of first farming groups of the Carpathian Basin. *Archaeol Anthropol Sci* **12**; 10.1007/s12520-020-01120-3 (2020).

8. Blanz, M., Balasse, M., Frémondeau, D., Gál, E., Osztás, A., Biller, A. Z., Nyerges, É. Á., Fiorillo, D., Bánffy, E. & Ivanova, M. Early Neolithic pastoral land use at Alsónyék-Bátaszék, Hungary (Starčevo culture): New insights from stable isotope ratios. *PloS one* **18,** e0295769; 10.1371/journal.pone.0295769 (2023).

9. Bánffy, E. *First farmers of the carpathian basin: changing patterns in subsistence, ritual and monumental figurines* (Oxbow Books, Oxford and Philadelphia, 2019).

10. Kempf, M. Take a seed! Revealing Neolithic landscape and agricultural development in the Carpathian Basin through multivariate statistics and environmental modelling. *PloS one* **16,** e0258206; 10.1371/journal.pone.0258206 (2021).

11. Gaastra, J. S., Greenfield, H. J. & Linden, M. V. Gaining traction on cattle exploitation: zooarchaeological evidence from the Neolithic Western Balkans. *Antiquity* **92,** 1462–1477; 10.15184/aqy.2018.178 (2018).

12. Evershed, R. P., Davey Smith, G., Roffet-Salque, M., Timpson, A., Diekmann, Y., Lyon, M. S., Cramp, L. J. E., Casanova, E., Smyth, J., Whelton, H. L., Dunne, J., Brychova, V., Šoberl, L., Gerbault, P., Gillis, R. E., Heyd, V., Johnson, E., Kendall, I., Manning, K., Marciniak, A., Outram, A. K., Vigne, J.-D., Shennan, S., Bevan, A., Colledge, S., Allason-Jones, L., Amkreutz, L., Anders, A., Arbogast, R.-M., Bălăşescu, A., Bánffy, E., Barclay, A., Behrens, A., Bogucki, P., Carrancho Alonso, Á., Carretero, J. M., Cavanagh, N., Claßen, E., Collado Giraldo, H., Conrad, M., Csengeri, P., Czerniak, L., Dębiec, M., Denaire, A., Domboróczki, L., Donald, C., Ebert, J., Evans, C., Francés-Negro, M., Gronenborn, D., Haack, F., Halle, M., Hamon, C., Hülshoff, R., Ilett, M., Iriarte, E., Jakucs, J., Jeunesse, C., Johnson, M., Jones, A. M., Karul, N., Kiosak, D., Kotova, N., Krause, R., Kretschmer, S., Krüger, M., Lefranc, P., Lelong, O., Lenneis, E., Logvin, A., Lüth, F., Marton, T., Marley, J., Mortimer, R., Oosterbeek, L., Oross, K., Pavúk, J., Pechtl, J., Pétrequin, P., Pollard, J., Pollard, R., Powlesland, D., Pyzel, J., Raczky, P., Richardson, A., Rowe, P., Rowland, S., Rowlandson, I., Saile, T., Sebők, K., Schier, W., Schmalfuß, G., Sharapova, S., Sharp, H., Sheridan, A., Shevnina, I., Sobkowiak-Tabaka, I., Stadler, P., Stäuble, H., Stobbe, A., Stojanovski, D., Tasić, N., van Wijk, I., Vostrovská, I., Vuković, J., Wolfram, S., Zeeb-Lanz, A. & Thomas, M. G. Dairying, diseases and the evolution of lactase persistence in Europe. *Nature* **608,** 336–345; 10.1038/s41586-022-05010-7 (2022).

13. Smyth, J., Gillis, R. E., Roffet-Salque, M., Johnson, E. V., Kendall, I. P., Krueger, M., Pyzel, J., Heyd, V., Marciniak, A., Vigne, J.-D., Balasse, M., Outram, A. K. & Evershed, R. P. Integrated approaches to understanding animal exploitation and dairying in the Central European Early Neolithic: a case study from Ludwinowo 7 (Kuyavia, Poland; c. 5250–5000 cal BC). *Front. Environ. Archaeol.* **2**; 10.3389/fearc.2023.1187087 (2023).

14. Halstead, P. Zooarchaeological evidence for livestock management in (earlier) Neolithic Europe: Outstanding questions and some limitations of current approaches. *Quaternary International* **683-684,** 42–50; 10.1016/j.quaint.2023.09.013 (2024).

15. Bánffy, E., Marton, T. & Osztás, A. Early neolithic settlement and burials at Alsónyék-Bátaszék. In *Neolithization of the Carpathian Basin: Northernmost distribution of the Starčevo/Körös culture,* edited by J. K. Kozłowski & P. Raczky (Polska Akademia Umiejętności, Kraków, 2010), pp. 37–51.
16. Oross, K., Osztás, A., Marton, T., Köhler, K., Ódor, J. G., Szécsényi-Nagy, A., Bánffy, E., Alt, K. W., Ramsey, C. B., Kromer, B., Bayliss, A., Hamilton, D. & Whittle, A. Midlife changes: the Sopot burial ground at Alsónyék. *Bericht der Römisch-Germanischen Kommission* **2013,** 151–178; 10.11588/berrgk.1938.0.37153 (2016).
17. Oross, K., Cramp, L. J., Gortva, G., Jakucs, J., Lyublyanovics, K., Marton, T., Serlegi, G., Vágvölgyi, B. & Whittle, A. 'It's still the same old story': The current southern Transdanubian approach to the Neolithisation process of central Europe. *Quaternary International* **560-561,** 154–178; 10.1016/j.quaint.2020.05.049 (2020).
18. Kalicz, N. The northern periphery of the Early Neolithic Starčevo culture in south-western Hungary: a case study of an excavation at Lake Balaton. *Documenta Praehistorica* **25,** 151–187 (1998).
19. Bánffy, E. & Whittle, A. Szentgyörgyvölgy-Pityerdomb and the formative phase of the LBK revisited. In *Wissensschichten : Festschrift für Wolfram Schier zu seinem 65. Geburtstag,* edited by E. Kaiser, M. Meyer, S. Scharl & S. Suhrbier (Verlag Marie Leidorf GmbH, Rahden/Westf., 2022).
20. Bánffy, E. *The 6th millennium BC boundary in Western Transdanubia and its role in the Central European neolithic transition: the Szentgyörgyvölgy-Pityerdomb settlement* (Archaeological Institute of the Hungarian Academy of Sciences, Budapest, 2004).
21. Jakucs, J., Bánffy, E., Oross, K., Voicsek, V., Bronk Ramsey, C., Dunbar, E., Kromer, B., Bayliss, A., Hofmann, D., Marshall, P. & Whittle, A. Between the Vinča and Linearbandkeramik Worlds: The Diversity of Practices and Identities in the 54th-53rd Centuries cal BC in Southwest Hungary and Beyond. *Journal of world prehistory* **29,** 267–336; 10.1007/s10963-016-9096-x (2016).
22. Milisauskas, S. & Kruk, J. Middle Neolithic/Early Copper Age, Continuity, Diversity, and Greater Complexity, 5500/5000-3500 BC. In *European Prehistory. Interdisciplinary Contributions to Archaeology,* edited by S. Milisauskas (Springer, New York, 2011).
23. Hernando, R., Gamarra, B., McCall, A., Cheronet, O., Fernandes, D., Sirak, K., Schmidt, R., Lozano, M., Szeniczey, T., Hajdu, T., Bárány, A., Kalli, A., Tutkovics, E. K., Köhler, K., Kiss, K., Koós, J., Csengeri, P., Király, Á., Horváth, A., Hajdu, M. L., Tóth, K., Patay, R., Feeney, R. N. M. & Pinhasi, R. Integrating buccal and occlusal dental microwear with isotope analyses for a complete paleodietary reconstruction of Holocene populations from Hungary. *Scientific Reports* **11,** 7034; 10.1038/s41598-021-86369-x (2021).
24. Kreuz, A., Pomázi, P. & Bánffy, E. Hungarian Neolithic landscapes, crops and diet – Signs of cultural decisions? *Quaternary International* **560-561,** 102–118; 10.1016/j.quaint.2020.06.008 (2020).

25. Mateiciucová, I. *Talking stones : the chipped stone industry in lower Austria and Moravia and the beginnings of the Neolithic in Central Europe (LBK), 5700-4900 BC* (Masarykova univerzita, Brno, 2008).

26. Bánffy, E. The beginnings of salt exploitation in the Carpathian basin (6th-5th millennium BC). *Documenta Praehistorica* **42**; 10.4312/dp.42.13 (2015).

27. Szécsényi-Nagy, A., Brandt, G., Haak, W., Keerl, V., Jakucs, J., Möller-Rieker, S., Köhler, K., Mende, B. G., Oross, K., Marton, T., Osztás, A., Kiss, V., Fecher, M., Pálfi, G., Molnár, E., Sebők, K., Czene, A., Paluch, T., Šlaus, M., Novak, M., Pećina-Šlaus, N., Ősz, B., Voicsek, V., Somogyi, K., Tóth, G., Kromer, B., Bánffy, E. & Alt, K. W. Tracing the genetic origin of Europe's first farmers reveals insights into their social organization. *Proceedings of the Royal Society B: Biological Sciences* **282**; 10.1098/rspb.2015.0339 (2015).

28. Marchi, N., Winkelbach, L., Schulz, I., Brami, M., Hofmanová, Z., Blöcher, J., Reyna-Blanco, C. S., Diekmann, Y., Thiéry, A., Kapopoulou, A., Link, V., Piuz, V., Kreutzer, S., Figarska, S. M., Ganiatsou, E., Pukaj, A., Struck, T. J., Gutenkunst, R. N., Karul, N., Gerritsen, F., Pechtl, J., Peters, J., Zeeb-Lanz, A., Lenneis, E., Teschler-Nicola, M., Triantaphyllou, S., Stefanović, S., Papageorgopoulou, C., Wegmann, D., Burger, J. & Excoffier, L. The genomic origins of the world's first farmers. *Cell* **185,** 1842-1859.e18; 10.1016/j.cell.2022.04.008 (2022).

29. Jakucs, J. LBK and Vinča in South-East Transdanubia: Comments on merging, interleaving and diversity. *Quaternary International* **560-561,** 119–141; 10.1016/j.quaint.2020.03.029 (2020).

30. Szécsényi-Nagy, A., Jakucs, J., Brandt, G., Bánffy, E. & Alt, K. W. Ancient DNA evidence for a homogeneous maternal gene pool in sixth millennium cal BC Hungary and the Central European LBK. In *Early farmers: the View from Archaeology and Science,* edited by A. Whittle & P. Bickle (Oxford University Press/British Academy, Oxford, United Kingdom, 2014), pp. 71–93.

31. Bánffy, E., Jakucs, J., Köhler, K., Marton, T., Oross, K. & Osztás, A. Buried in mud, buried in clay: specially arranged settlement burials from in and around the Danubian Sárköz, Neolithic southern Hungary. Chapter 5. In *The Neolithic of Europe. Papers in honour of Alasdair Whittle,* edited by P. Bickle, V. Cummings, D. Hofmann & J. Pollard (Oxbow Books, Oxford and Philadelphia, 2017), pp. 47–61.

32. Barna, J. P. *The Formation of the Lengyel Culture in South-Western Transdanubia* (Archaeopress, Budapest, 2017).

33. Kalicz, N. Der neuere Forschungsstand über die Lengyel Kultur. In *Sites and Stones: Lengyel Culture in Western Hungary and Beyond. A Review of the Current Research. Lengyel '99 and IGCP-442 Conference, Veszprém, 1999,* edited by J. Regenye (Viza Press, Veszprém, 2001), pp. 7–13.

34. Marton, T. & Oross, K. Siedlungsforschung in linearbandkeramischen Fundorten in Zentral- und Südtransdanubien - Wiege, Peripherie oder beides? In *Siedlungsstruktur und Kulturwandel in der Bandkeramik. Beiträge der internationalen Tagung "Neue Fragen zur*

Bandkeramik oder alles beim Alten?!", Leipzig, 23. bis 24. September 2010, edited by R. Smolnik, S. Wolfram & H. Stäuble (LfA Sachsen, Dresden, 2012), pp. 220–239.

35. Oross, K., Simmer, L. & Staub, P. Regionality in fluidity: the Linearbandkeramik site at Keszthely-Lendl Adolf út in western Hungary and its hinterland. In *"Trans Lacum Pelsonem". Prähistorische Forschungen in Südwestungarn (5500-500 v. Chr.)/Prehistoric Research in South-Western Hungary (5500-500 BC),* edited by E. Bánffy & J. P. Barna (Marie Leidorf GmbH, Rahden/Westf., 2019), pp. 9–72.

36. Regenye, J. A lengyeli kultúra újabb lelőhelyei Veszprém megyében. *A Veszprém Megyei Múzeumok Közleményei,* 9–19 (2000).

37. Raczky, P. The Tisza culture of the Great Hungarian Plain. Referate der Teilnehmer am Internationalen Symposium über die "Rolle des Schwarzen Meeres in der Urgeschichte Europas", Dobriæ 1988. *Studia praehistorica,* 162–176 (1992).

38. Kalicz, N. Wenden des Spätneolithikums im Oberen Theißgebiet (Fordulatok a Felső-Tisza-vidék késő neolitikumában). *Jósa András Múzeumtörténeti évkönyv* **36,** 263–290 (1994).

39. Füzesi, A., Rassmann, K., Bánffy, E. & Raczky, P. Human Activities on a Late Neolithic Tell-like Settlement Complex of the Hungarian Plain (Öcsöd-Kováshalom). Chap. 10. In *Current Approaches to Tells in the Prehistoric Old World,* edited by A. BLANCO-GONZÁLEZ & T. L. KIENLIN (Oxbow Books2020), pp. 139–162.

40. Bartosiewicz, L. Plain talk: animals, environment and culture in the Neolithic of the Carpathian Basin and adjacent areas. In *(Un)settling the Neolithic: Breaking down concepts, boundaries and origins,* edited by D. W. Bailey, A. Whittle & V. Cummings (Oxbow Books, Oxford, 2005), pp. 51–63.

41. Hoekman-Sites, H. A. & Giblin, J. I. Prehistoric animal use on the Great Hungarian Plain: A synthesis of isotope and residue analyses from the Neolithic and Copper Age. *Journal of Anthropological Archaeology* **31,** 515–527; 10.1016/j.jaa.2012.05.002 (2012).

42. Giblin, J. I. Strontium isotope analysis of Neolithic and Copper Age populations on the Great Hungarian Plain. *Journal of Archaeological Science* **36,** 491–497; 10.1016/j.jas.2008.09.034 (2009).

43. Giblin, J. I., Knudson, K. J., Bereczki, Z., Pálfi, G. & Pap, I. Strontium isotope analysis and human mobility during the Neolithic and Copper Age: a case study from the Great Hungarian Plain. *Journal of Archaeological Science* **40,** 227–239; 10.1016/j.jas.2012.08.024 (2013).

44. Depaermentier, M. L. C., Kempf, M., Bánffy, E. & Alt, K. W. Tracing mobility patterns through the 6th-5th millennia BC in the Carpathian Basin with strontium and oxygen stable isotope analyses. *PloS one* **15,** e0242745; 10.1371/journal.pone.0242745 (2020).

45. Gulyás, S. & Sümegi, P. Farming and/or foraging? New environmental data to the life and economic transformation of Late Neolithic tell communities (Tisza Culture) in SE Hungary. *Journal of Archaeological Science* **38,** 3323–3339; 10.1016/j.jas.2011.07.019 (2011).

46. Parkinson, W. A., Yerkes, R. W., Gyucha, A., Sarris, A., Morris, M. & Salisbury, R. B. Early Copper Age Settlements in the Körös Region of the Great Hungarian Plain. *Journal of Field Archaeology,* 164–183 (2010).

47. Gyucha, A. *Prehistoric village social dynamics. The Early Copper Age in the Körös region* (Archaeolingua Alapítvány, Budapest, 2015).

48. Giblin, J. I. & Yerkes, R. W. Diet, dispersal and social differentiation during the Copper Age in eastern Hungary. *Antiquity* **90,** 81–94; 10.15184/aqy.2016.3 (2016).

49. Regenye, J., Oross, K., Bánffy, E., Dunbar, E., Friedrich, R., Bayliss, A., Beavan, N., Gaydarska, B. & Whittle, A. Some Balaton-Lasinja Graves from Veszprém-Jutasi Út and an Outline Chronology for the Earlier Copper Age in Western Hungary. *Documenta Praehistorica* **49,** 280–299; 10.4312/dp.49.4 (2022).

50. Magyari, E. K., Raczky, P., Merkl, M., Pálfi, I., Darabos, G., Hajnalova, M. & Moskal-Hoyo, M. Review on vegetation, landscape and climate changes in the Carpathian Basin during the Neolithic and Chalcolithic period. *Vegetation History and Archaeobotany*; 10.1007/s00334-024-00986-w (2024).

51. Ács, F., Breuer, H. & Skarbit, N. Climate of Hungary in the twentieth century according to Feddema. *Theoretical and Applied Climatology* **119,** 161–169; 10.1007/s00704-014-1103-5 (2015).

52. Demény, A., Czuppon, G., Siklósy, Z., Leél-Őssy, S., Lin, K., Shen, C.-C. & Gulyás, K. Mid-Holocene climate conditions and moisture source variations based on stable H, C and O isotope compositions of speleothems in Hungary. *Quaternary International* **293,** 150–156; 10.1016/j.quaint.2012.05.035 (2013).

53. Kiss, T., Hernesz, P., Sümeghy, B., Györgyövics, K. & Sipos, G. The evolution of the Great Hungarian Plain fluvial system – Fluvial processes in a subsiding area from the beginning of the Weichselian. *Quaternary International* **388,** 142–155; 10.1016/j.quaint.2014.05.050 (2015).

54. Jakab, G., Majkut, P., Juhász, I., Gulyás, S., Sümegi, P. & Törőcsik, T. Palaeoclimatic signals and anthropogenic disturbances from the peatbog at Nagybárkány (North Hungary). In *Palaeolimnological Proxies as Tools of Environmental Reconstruction in Fresh Water,* edited by K. Buczkó, J. Korponai, J. Padisák & S. W. Starratt (Springer Netherlands, Dordrecht, 2009), pp. 87–106.

55. Gardner, A. R. Neolithic to Copper Age woodland impacts in northeast Hungary? Evidence from the pollen and sediment chemistry records. *The Holocene* **12,** 541–553; 10.1191/0959683602hl561rp (2002).

56. Novothny, Á., Frechen, M. & Horváth, E. Luminescence dating of periods of sand movement from the Gödöllő Hills, Hungary. *Geomorphology* **122,** 254–263; 10.1016/j.geomorph.2010.04.013 (2010).

57. Magyari, E. K., Chapman, J. C., Passmore, D. G., Allen, J., Huntley, J. P. & Huntley, B. Holocene persistence of wooded steppe in the Great Hungarian Plain. *Journal of Biogeography* **37,** 915–935; 10.1111/j.1365-2699.2009.02261.x (2010).

58. Uj, B., Nagy, A., Saláta, D., Laborczi, A., Malatinszky, Á., Bakó, G., Danyik, T., Tóth, A., S. Falusi, E., Gyuricza, C., Póti, P. & Penksza, K. Wetland habitats of the Kis-Sárrét 1860–2008 (Körös-Maros National Park, Hungary). *Journal of Maps* **12,** 211–221; 10.1080/17445647.2014.1001799 (2016).

59. Willis, K. J., Braun, M., Sumegi, P. & Toth, A. Does Soil Change Cause Vegetation Change or Vice Versa? A Temporal Perspective from Hungary. *Ecology* **78,** 740; 10.2307/2266054 (1997).

60. Willis, K. J., Rudner, E. & Sümegi, P. The Full-Glacial Forests of Central and Southeastern Europe. *Quaternary Research* **53,** 203–213; 10.1006/qres.1999.2119 (2000).

61. Magyari, E. K. Late Quaternary vegetation history in the Hortobágy steppe and Middle Tisza floodplain, NE Hungary. *Studia botanica hungarica,* 185–203 (2011).

62. Hertelendi, E., Sümegi, P. & Szöör, G. Geochronologic and Paleoclimatic Characterization of Quaternary Sediments in the Great Hungarian Plain. *Radiocarbon* **34,** 833–839; 10.1017/S0033822200064146 (1992).

63. Magyari, E., Sümegi, P., Braun, M., Jakab, G. & Molnár, M. Retarded wetland succession: anthropogenic and climatic signals in a Holocene peat bog profile from north-east Hungary. *Journal of Ecology* **89,** 1019–1032; 10.1111/j.1365-2745.2001.00624.x (2001).

64. Hedges, R. E. M., Bentley, R. A., Bickle, P., Cullen, P., Dale, C., Fibiger, L., Hamilton, J., Hofmann, D., Nowell, G. & Whittle, A. The supra-regional approach. In *The first farmers of central Europe. Diversity in LBK lifeways,* edited by P. Bickle & A. Whittle (Oxbow Books and the David Brown Book Company, Oakville, CT, 2013), pp. 343–384.

65. Kercsmár, Z., Budai, T., Csillag, G., Selmeczi, I. & Sztanó, O. (eds.). *Surface geology of Hungary. Explanatory notes to the geological map of Hungary (1:500 000)* (Geological and Geophysical Institute of Hungary, Budapest, 2015).

66. Timár, G., Sümegi, P. & Horváth, F. Late Quaternary dynamics of the Tisza River: Evidence of climatic and tectonic controls. *Tectonophysics* **410,** 97–110; 10.1016/j.tecto.2005.06.010 (2005).

67. Moskal-del Hoyo, M., Lityńska-Zając, M., Raczky, P., Anders, A. & Magyari, E. K. The character of the Atlantic oak woods of the Great Hungarian Plain. *Quaternary International* **463,** 337–351; 10.1016/j.quaint.2017.02.029 (2018).

68. Kiss, T., Sümeghy, B. & Sipos, G. Late Quaternary paleodrainage reconstruction of the Maros River alluvial fan. *Geomorphology* **204,** 49–60; 10.1016/j.geomorph.2013.07.028 (2014).

69. Kasse, C., Bohncke, S., Vandenberghe, J. & Gábris, G. Fluvial style changes during the last glacial–interglacial transition in the middle Tisza valley (Hungary). *Proceedings of the Geologists' Association* **121,** 180–194; 10.1016/j.pgeola.2010.02.005 (2010).

70. Laborczi, A., Szatmári, G., Takács, K. & Pásztor, L. Mapping of topsoil texture in Hungary using classification trees. *Journal of Maps* **12,** 999–1009; 10.1080/17445647.2015.1113896 (2016).

71. Dobos, E., Micheli, E., Baumgardner, M. F., Biehl, L. & Helt, T. Use of combined digital elevation model and satellite radiometric data for regional soil mapping. *Geoderma* **97,** 367–391; 10.1016/S0016-7061(00)00046-X (2000).

72. Schofield, R., Thomas, D. S. G. & Kirkby, M. J. Causal processes of soil salinization in Tunisia, Spain and Hungary. *Land Degradation & Development* **12,** 163–181; 10.1002/ldr.446 (2001).

73. Tóth, T., Kuti, L., Kabos, L. & Pásztor, L. Use of Digitalized Hydrogeological Maps for Evaluation of Salt-Affected Soils of Large Areas. *Arid Land Research and Management* **15,** 329–346; 10.1080/153249801753127624 (2001).

74. Mádl-Szőnyi, J., Tóth, J. & Pogácsás, G. Soil and wetland salinization in the framework of the Danube-Tisza Interfluve hydrogeologic type section. *Central European Geology* **51,** 203–217; 10.1556/ceugeol.51.2008.3.3 (2008).

75. Wang, T., Wei, D., Chang, X., Yu, Z., Zhang, X., Wang, C., Hu, Y. & Fuller, B. T. Tianshanbeilu and the Isotopic Millet Road: reviewing the late Neolithic/Bronze Age radiation of human millet consumption from north China to Europe. *National science review* **6,** 1024–1039; 10.1093/nsr/nwx015 (2019).

76. Filipović, D., Meadows, J., Corso, M. D., Kirleis, W., Alsleben, A., Akeret, Ö., Bittmann, F., Bosi, G., Ciută, B., Dreslerová, D., Effenberger, H., Gyulai, F., Heiss, A. G., Hellmund, M., Jahns, S., Jakobitsch, T., Kapcia, M., Klooß, S., Kohler-Schneider, M., Kroll, H., Makarowicz, P., Marinova, E., Märkle, T., Medović, A., Mercuri, A. M., Mueller-Bieniek, A., Nisbet, R., Pashkevich, G., Perego, R., Pokorný, P., Pospieszny, Ł., Przybyła, M., Reed, K., Rennwanz, J., Stika, H.-P., Stobbe, A., Tolar, T., Wasylikowa, K., Wiethold, J. & Zerl, T. New AMS 14C dates track the arrival and spread of broomcorn millet cultivation and agricultural change in prehistoric Europe. *Sci Rep* **10,** 13698; 10.1038/s41598-020-70495-z (2020).

77. Alt, K. W., Knipper, C., Peters, D., Müller, W., Maurer, A.-F., Kollig, I., Nicklisch, N., Müller, C., Karimnia, S., Brandt, G., Roth, C., Rosner, M., Mende, B., Schöne, B. R., Vida, T. & Freeden, U. von. Lombards on the move--an integrative study of the migration period cemetery at Szólád, Hungary. *PloS one* **9,** e110793; 10.1371/journal.pone.0110793 (2014).

78. Motuzaite-Matuzeviciute, G., Staff, R. A., Hunt, H. V., Liu, X. & Jones, M. K. The early chronology of broomcorn millet (Panicum miliaceum) in Europe. *Antiquity* **87,** 1073–1085; 10.1017/S0003598X00049875 (2013).

79. Pospieszny, Ł., Makarowicz, P., Lewis, J., Górski, J., Taras, H., Włodarczak, P., Szczepanek, A., Ilchyshyn, V., Jagodinska, M. O., Czebreszuk, J., Muzolf, P., Nowak, M., Polańska, M., Juras, A., Chyleński, M., Wójcik, I., Lasota-Kuś, A., Romaniszyn, J., Tunia, K., Przybyła, M. M., Grygiel, R., Matoga, A., Makowiecki, D. & Goslar, T. Isotopic evidence of millet consumption in the Middle Bronze Age of East-Central Europe. *Journal of Archaeological Science* **126,** 105292; 10.1016/j.jas.2020.105292 (2021).

80. Whittle, A., Bentley, R. A., Cramp, L. J. E., Domboróczki, L., Hamilton, J., Hedges, R. E. M., Kalicz, N., Kovács, Z. E., Marton, T., Oross, K. & Raczky, P. Hungary. In *The first*

farmers of central Europe. Diversity in LBK lifeways, edited by P. Bickle & A. Whittle (Oxbow Books and the David Brown Book Company, Oakville, CT, 2013), pp. 49–100.

81. Doppler, T., Gerling, C., Heyd, V., Knipper, C., Kuhn, T., Lehmann, M. F., Pike, A. W. & Schibler, J. Landscape opening and herding strategies: Carbon isotope analyses of herbivore bone collagen from the Neolithic and Bronze Age lakeshore site of Zurich-Mozartstrasse, Switzerland. *Quaternary International* **436,** 18–28; 10.1016/j.quaint.2015.09.007 (2017).

82. Drucker, D. G., Bridault, A., Hobson, K. A., Szuma, E. & Bocherens, H. Can carbon-13 in large herbivores reflect the canopy effect in temperate and boreal ecosystems? Evidence from modern and ancient ungulates. *Palaeogeography, Palaeoclimatology, Palaeoecology* **266,** 69–82; 10.1016/j.palaeo.2008.03.020 (2008).

83. Domboróczki, L. Neolithization in northeastern Hungary: old theories and new perspectives. In *Die Neolithisierung Mitteleuropas: = The Spread of the Neolithic to Central Europe,* edited by D. Gronenborn & J. Petrasch (Verlag des Römisch-Germanischen Zentralmuseums, Mainz, 2010), pp. 175–184.

84. Nyerges, É. Á. & Biller, A. Z. Neolithic animal husbandry in the Tolnai-Sárköz Region on the basis of the archaeozoological finds from the Alsónyék–Bátaszék archaeological site. *Hungarian Archaeology E-Journal,* 1–7 (2015).
