# Supplementary figures and images for: Cultural diversity shaped neolithic subsistence in the Carpathian Basin
Source: Sci Rep. 2025 Feb 4;15:4281. doi: 10.1038/s41598-025-88541-z (PMC11794553; doi:10.1038/s41598-025-88541-z)

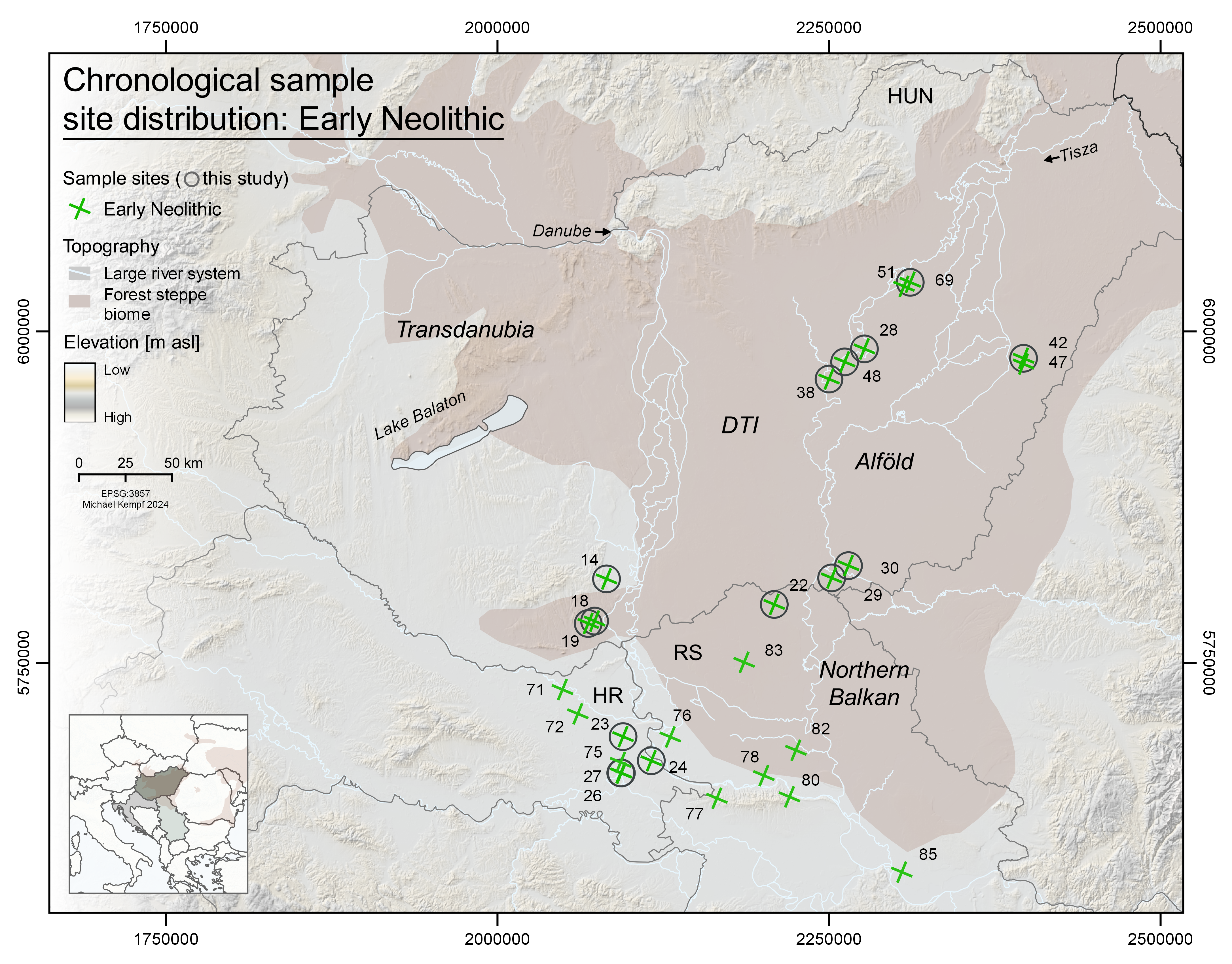

Supplement: Supplementary file 8 — Supplementary Material 8 [file 41598_2025_88541_MOESM8_ESM.tif]

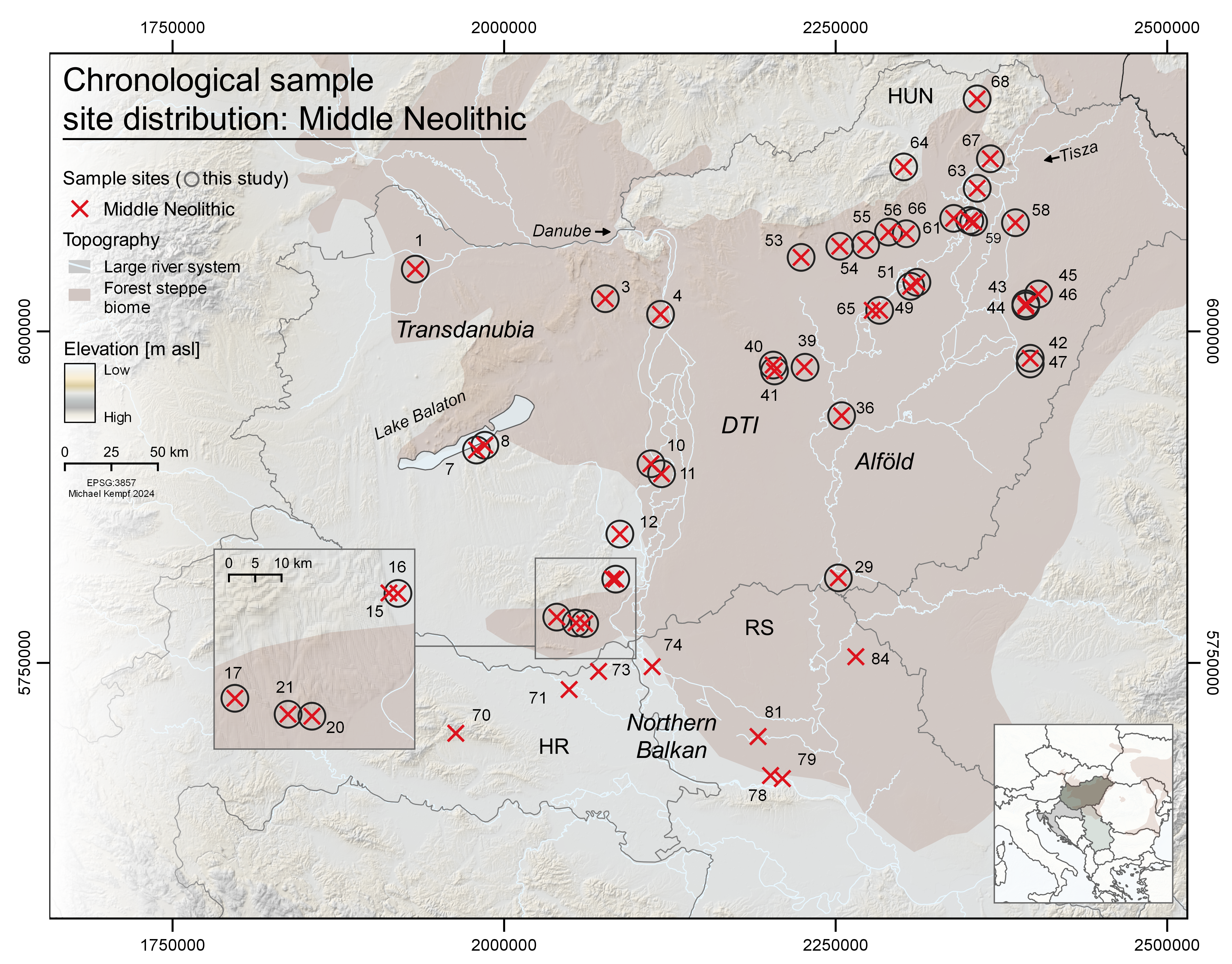

Supplement: Supplementary file 9 — Supplementary Material 9 [file 41598_2025_88541_MOESM9_ESM.tif]

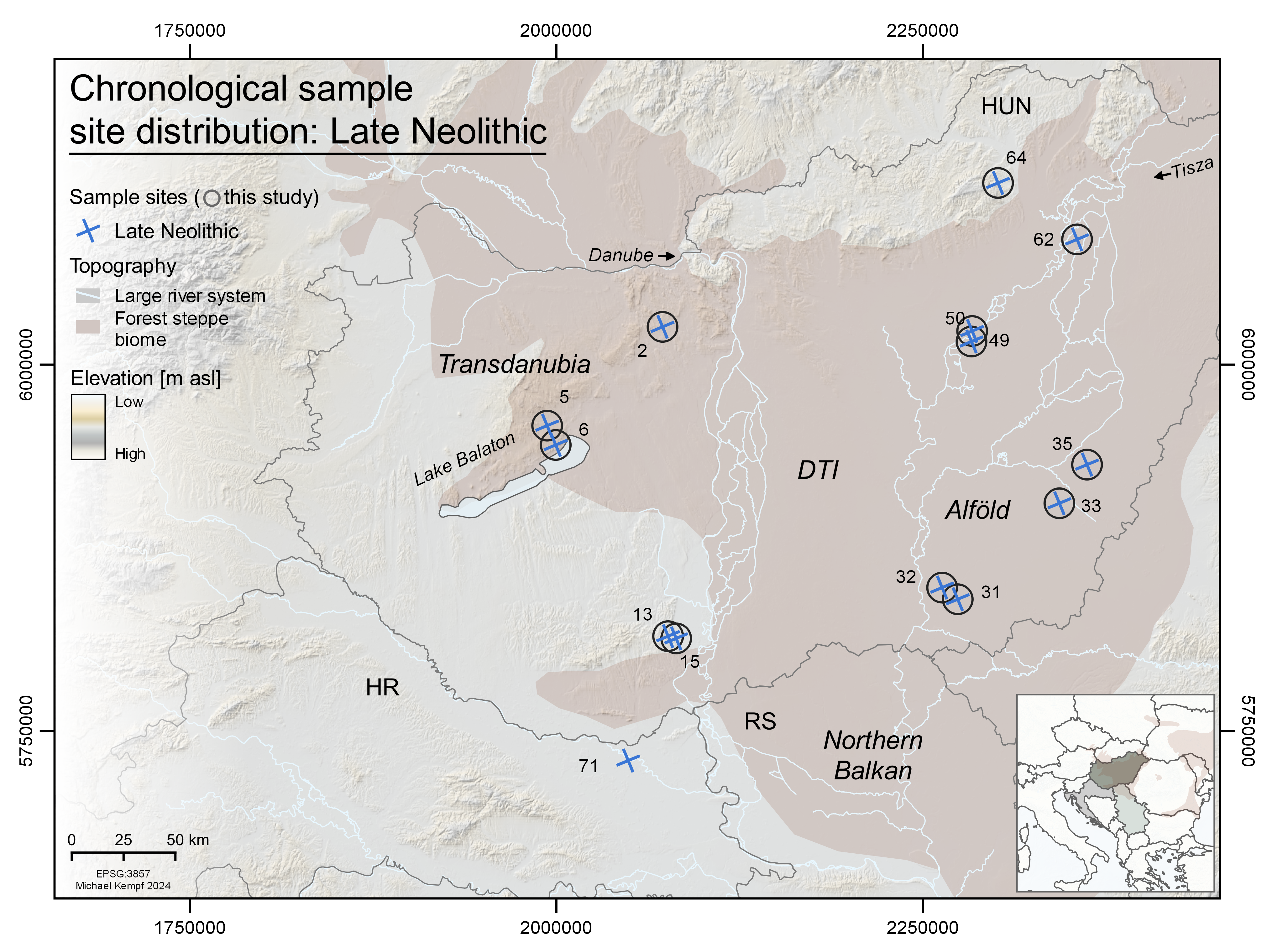

Supplement: Supplementary file 10 — Supplementary Material 10 [file 41598_2025_88541_MOESM10_ESM.tif]

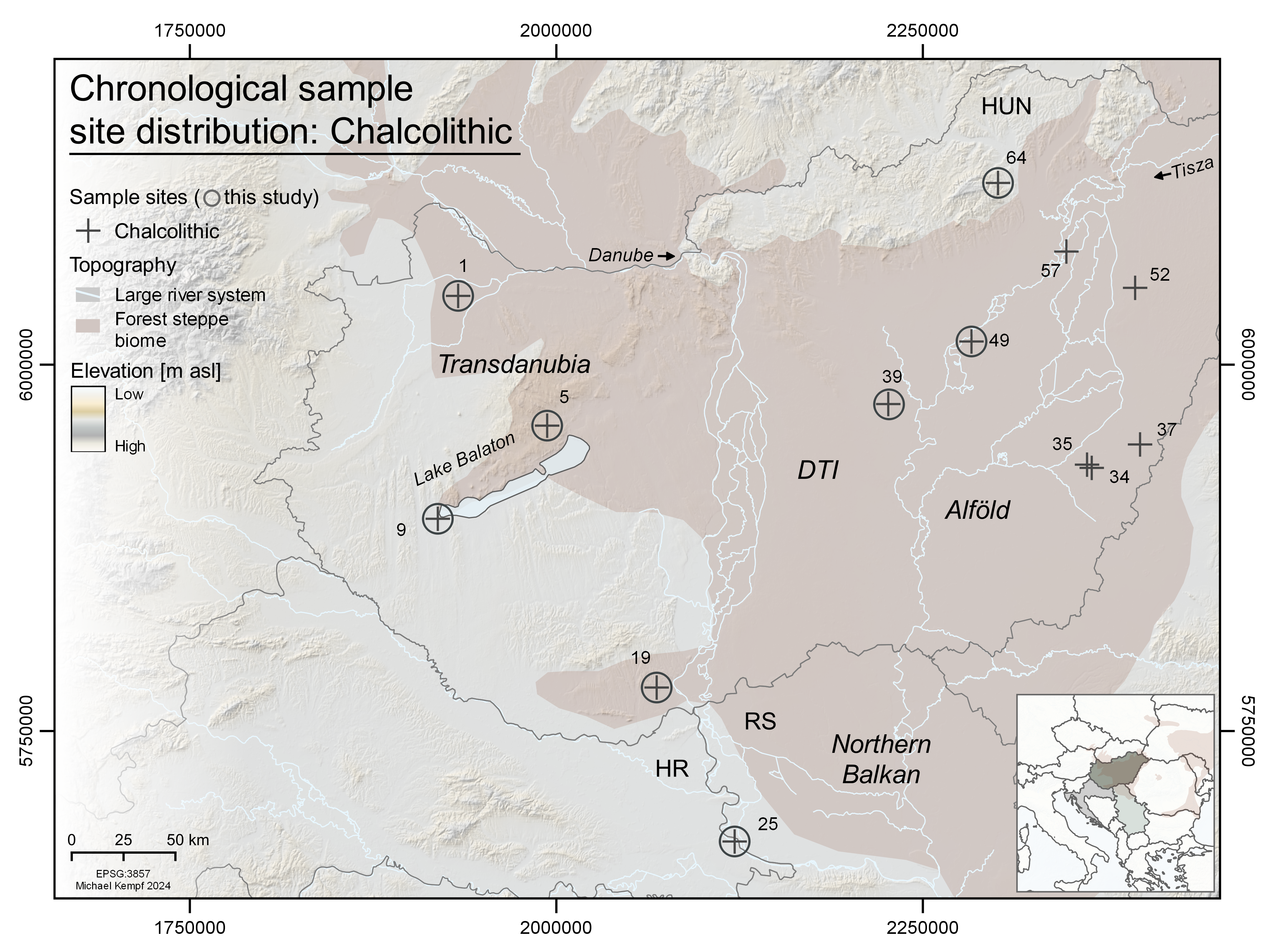

Supplement: Supplementary file 11 — Supplementary Material 11 [file 41598_2025_88541_MOESM11_ESM.tif]

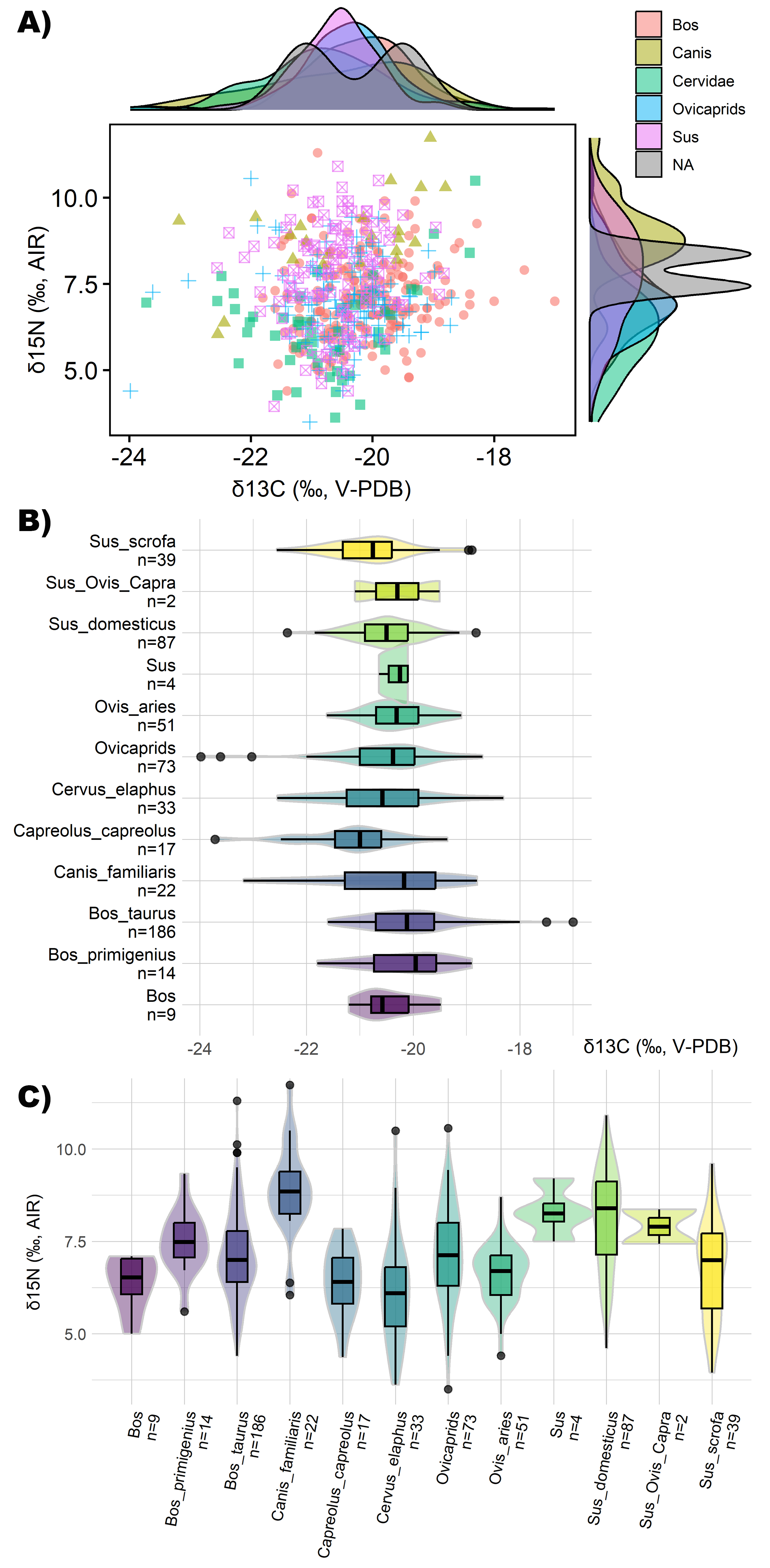

Supplement: Supplementary file 12 — Supplementary Material 12 [file 41598_2025_88541_MOESM12_ESM.tif]

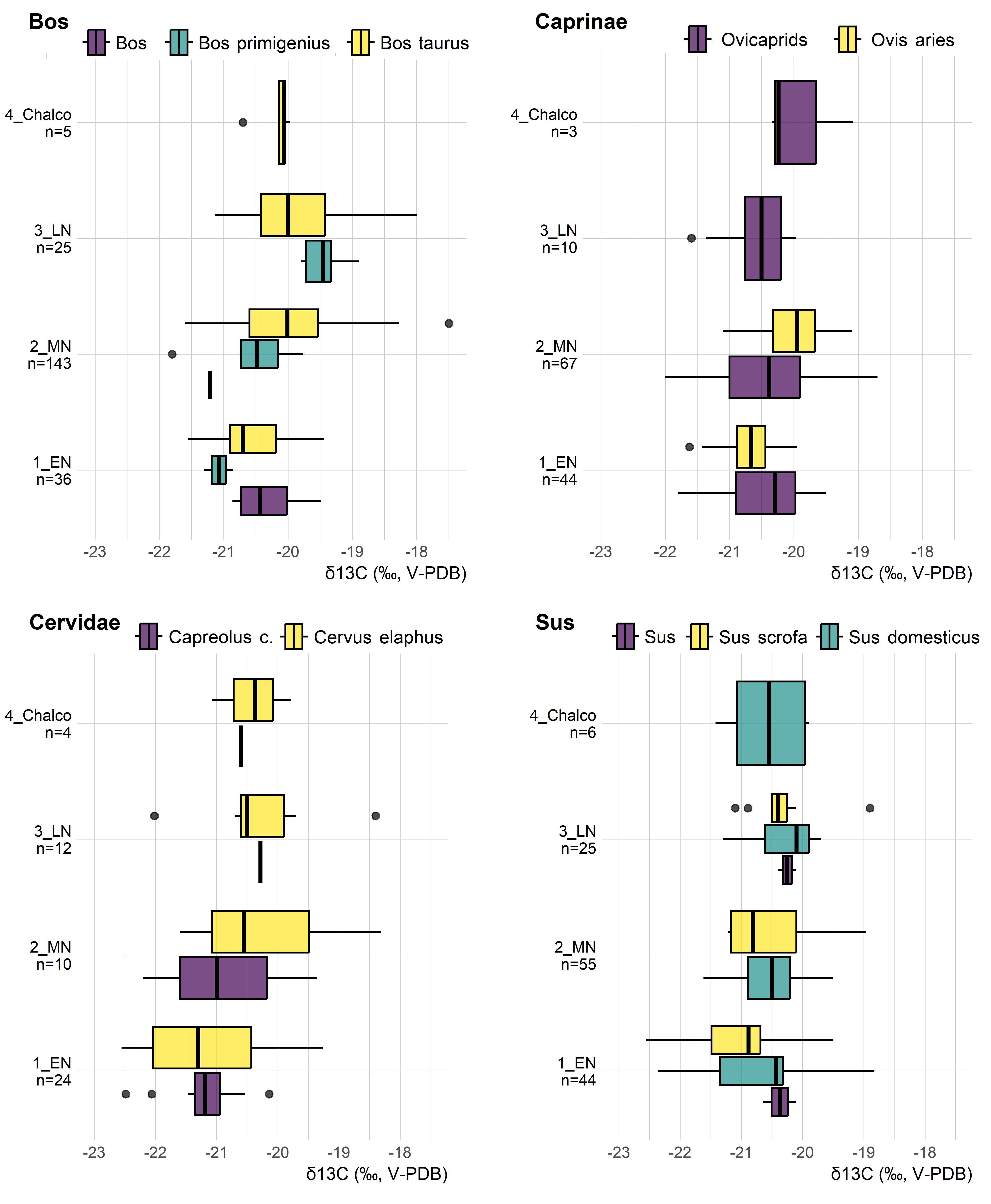

Supplement: Supplementary file 13 — Supplementary Material 13 [file 41598_2025_88541_MOESM13_ESM.tif]

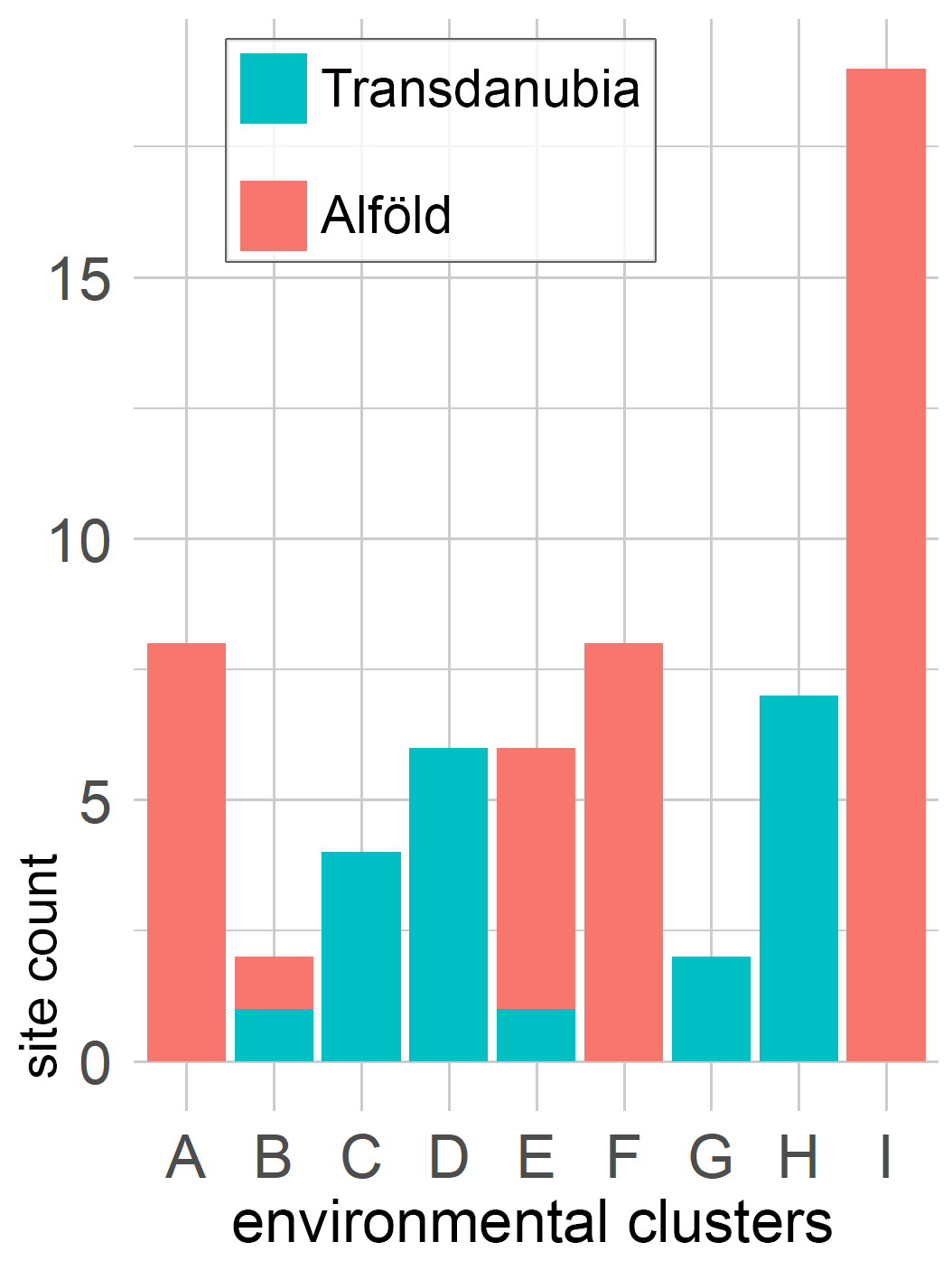

Supplement: Supplementary file 14 — Supplementary Material 14 [file 41598_2025_88541_MOESM14_ESM.tif]

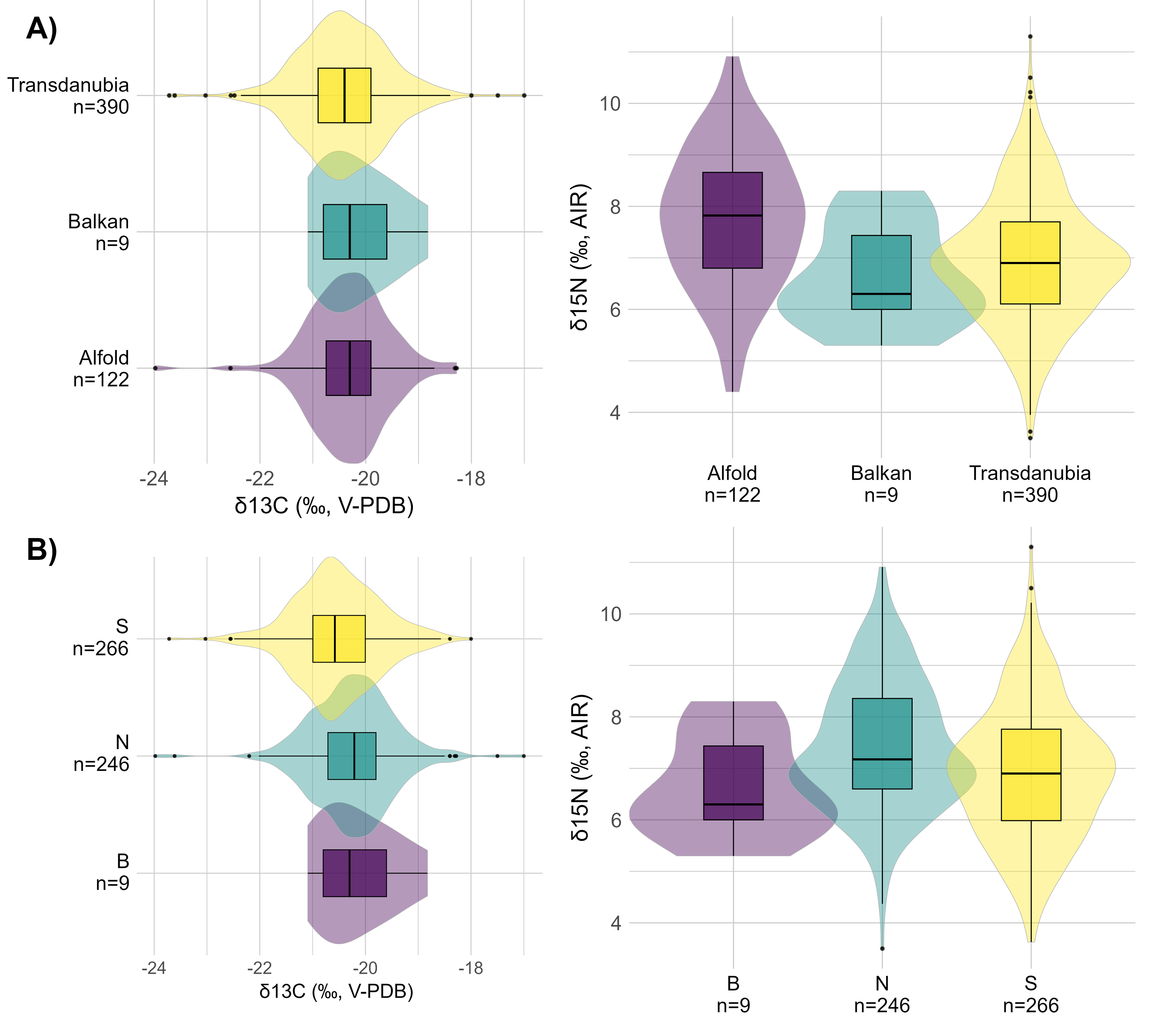

Supplement: Supplementary file 16 — Supplementary Material 16 [file 41598_2025_88541_MOESM16_ESM.tif]

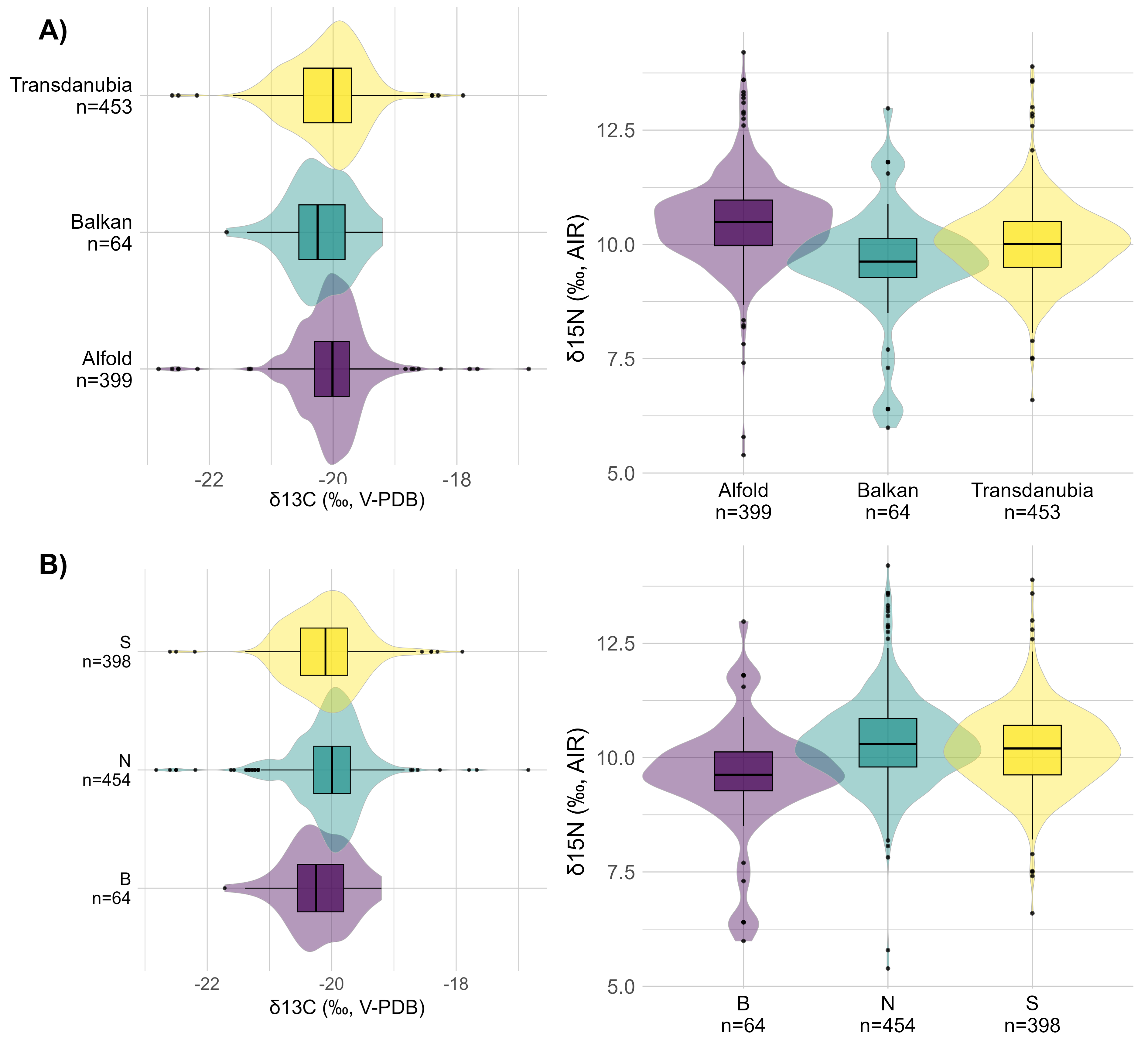

Supplement: Supplementary file 17 — Supplementary Material 17 [file 41598_2025_88541_MOESM17_ESM.tif]

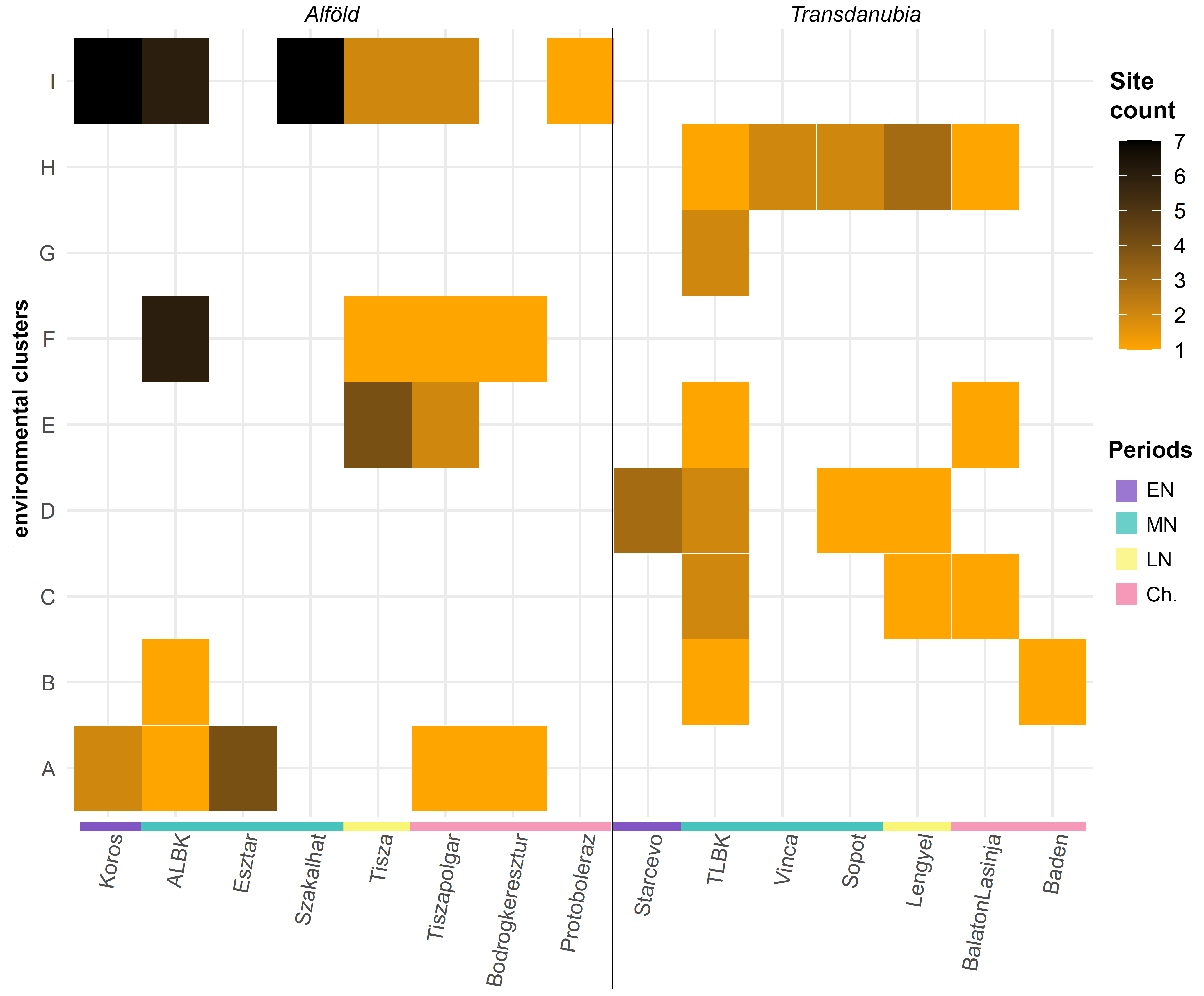

Supplement: Supplementary file 19 — Supplementary Material 19 [file 41598_2025_88541_MOESM19_ESM.tif]

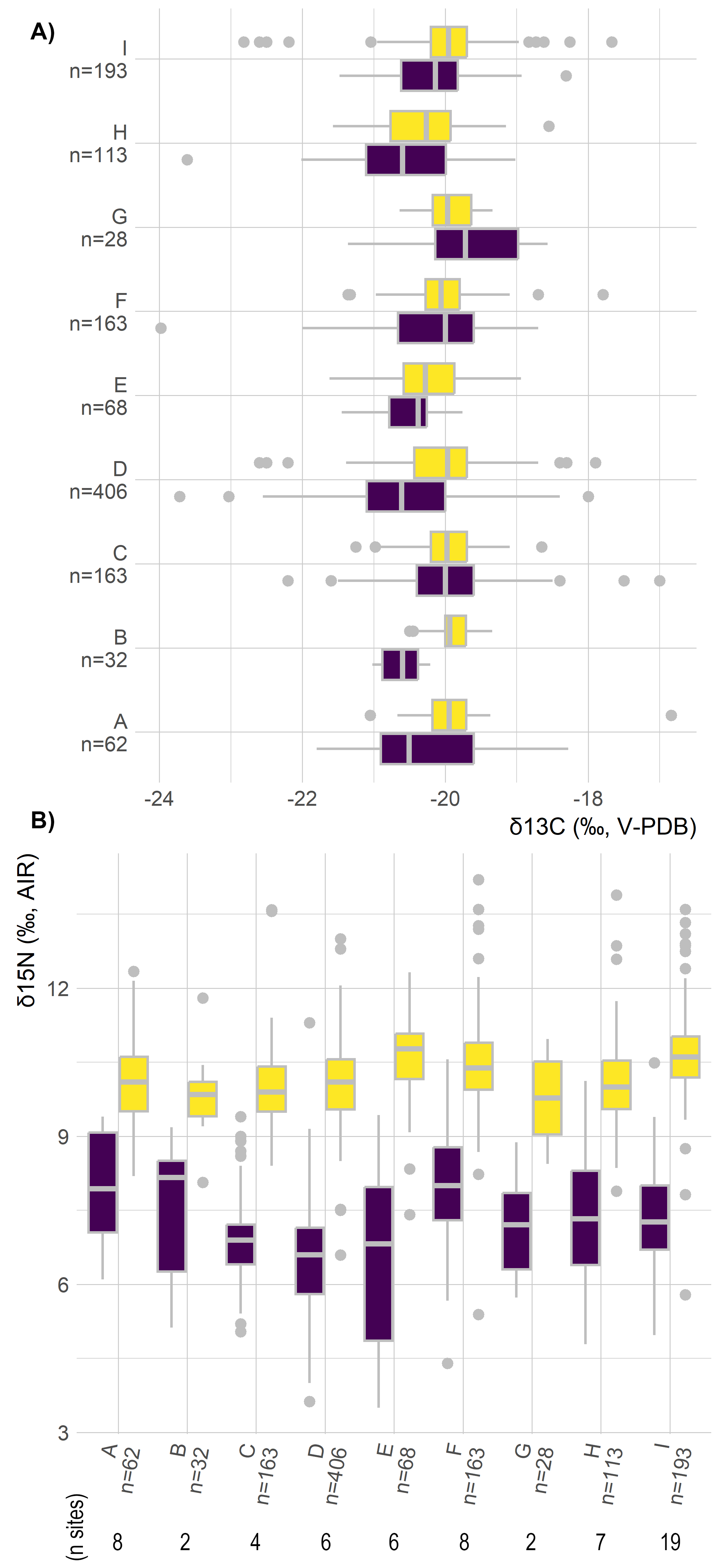

Supplement: Supplementary file 20 — Supplementary Material 20 [file 41598_2025_88541_MOESM20_ESM.tif]
